# From Gas Chromatography–Mass Spectrometry (GC–MS) to Network Pharmacology: System-Level Insights into the Multi-Target Biological Potential of *Flaveria trinervia* (Spreng.) C. Mohr

**DOI:** 10.3390/cimb48020160

**Published:** 2026-02-01

**Authors:** Christopher Torres Flores, Eduardo Pérez-Campos, Laura Pérez-Campos Mayoral, Luis Ángel Laguna-Barrios, Karen Beatriz Méndez-Rodríguez, Francisco Javier Pérez-Vázquez, Eduardo Pérez Campos-Mayoral, Carlos Mauricio Lastre-Domínguez, Efrén Emmanuel Jarquín González, Margarito Martínez Cruz, María del Socorro Pina Canseco, Zoila Mora Guzmán, Karol Celeste López Montesinos, Hector A. Cabrera-Fuentes, María Teresa Hernández-Huerta

**Affiliations:** 1 Centro de Investigación Facultad de Medicina UNAM-UABJO, Facultad de Medicina y Cirugía, Universidad Autónoma “Benito Juárez” de Oaxaca, Oaxaca 68020, Mexico; chris.tf@hotmail.com (C.T.F.); lperezcampos.fmc@uabjo.mx (L.P.-C.M.); labl890629.fmc@uabjo.mx (L.Á.L.-B.); eperezcampos.fmc@uabjo.mx (E.P.C.-M.); mpina.cat@uabjo.mx (M.d.S.P.C.); zmora.fmc@uabjo.mx (Z.M.G.); 2 División de Estudios de Posgrado e Investigación, Tecnológico Nacional de México/Instituto Tecnológico de Oaxaca, Oaxaca 68030, Mexico; pcampos@itoaxaca.edu.mx (E.P.-C.); carlos.lastre@itoaxaca.edu.mx (C.M.L.-D.); mcruz@itoaxaca.edu.mx (M.M.C.); 3 Coordinación de Innovación y Aplicación de la Ciencia y la Tecnología, Universidad Autónoma de San Luis Potosí, San Luis Potosí 78210, Mexico; beatriz.mendez@uaslp.mx (K.B.M.-R.); javier.perez@uaslp.mx (F.J.P.-V.); 4 Dirección General de los Servicios de Salud de Oaxaca, Secretaria de Salud, Servicios de Salud de Oaxaca, Oaxaca 68000, Mexico; drefrenjg@icloud.com; 5 Dirección de la División de Investigación y Desarrollo Científico, Benemérita Universidad de Oaxaca, Oaxaca 68000, Mexico; karolc1200@gmail.com; 6 R&D Group, Vice Presidency Scientific Research & Innovation, Imam Abdulrahman bin Faisal University (IAU), Dammam 31451, Saudi Arabia; 7 División de Estudios de Posgrado e Investigación, Tecnológico Nacional de México/Instituto Tecnológico de Tijuana, Tijuana 22414, Mexico; 8 Secretaría de Ciencia, Humanidades, Tecnología e Innovación (SECIHTI), Facultad de Medicina y Cirugía, Universidad Autónoma “Benito Juárez” de Oaxaca, Oaxaca 68020, Mexico

**Keywords:** *Flaveria trinervia*, gas chromatography–mass spectrometry, network pharmacology, bioactive metabolites, tumor, immune disorders, plant extracts, pharmaceutical research

## Abstract

*Flaveria trinervia* (Spreng) C. Mohr is a plant traditionally used in Mexican medicine. In this study, gas chromatography–mass spectrometry (GC–MS) combined with network pharmacology was employed to characterize volatile and semi-volatile metabolites from *F. trinervia* leaves and to explore their potential system-level mechanisms of action in inflammatory and tumor-related disorders. A dual extraction strategy (hexane/dichloromethane and acetone/chloroform) was applied, followed by GC–MS-based compound identification. Putative molecular targets were predicted using established pharmacological databases, and protein–protein interaction networks were constructed to identify topological features and enriched biological pathways. A total of 11 bioactive compounds were tentatively identified with an identity level of ≥80%, with seven shared between both extracts, including phytol, germacrene D, caryophyllene oxide, pinene isomers, squalene, and 2,2′:5′,2″-terthiophene, metabolites previously reported to exhibit antioxidant, anti-inflammatory, and cytotoxic activities. Network topology analysis identified ESR1, RXRA/B/G, NCOA2, and CYP19A1 as central nodes, reflecting convergence on signaling axes involved in apoptosis, cell proliferation, immune modulation, and transcriptional regulation pathways. Functional enrichment analysis revealed significant associations with KEGG pathways related to immune modulation, neuroendocrine regulation, and cancer-associated pathways. Collectively, these findings suggest a multitarget biological and multipathway pharmacological profile for *F. trinervia*, consistent with previously reported biological activities. The concordance between in silico predictions and existing experimental evidence strengthens the pharmacological relevance of the identified metabolites and supports their prioritization for further experimental validation, including mechanistic and pharmacokinetic studies, in metabolic, immune, neurological, and cancer-related contexts.

## 1. Introduction

Natural products and plant extracts are a key source of bioactive compounds, also known as phytoconstituents, in pharmaceutical research, playing a fundamental role in the discovery of new drugs. Their therapeutic potential has been investigated for the prevention and treatment of various human diseases [1,2], including different types of cancer and immune disorders, which are global health problems that require new therapeutic strategies.

*Flaveria trinervia* (Spreng.) C. Mohr (*F. trinervia*), also known as “Scotch broom [3] or Clustered yellowtops” [4], is a plant native to Mexico used in traditional medicine for the treatment of diseases such as diarrhea [5], dysentery [6], parasitosis [7], gastritis, and respiratory diseases [8]. In addition, some studies have demonstrated the hepatoprotective [9], prophylactic [10], analgesic, anti-inflammatory [11], antioxidant, and cytotoxic [12] effects of *F. trinervia*. These biological activities have been attributed to a diverse array of bioactive phytoconstituents, including: ergosterol, octadecanoic acid, *n*-hexadecanoic acid, tetradecanoic acid, isopropyl palmitate, octacosyl heptafluorobutyrate, pentafluoropropionic acid tridecyl ester, tetratriacontyl trifluoroacetate, propanoic acid derivatives, 3-deoxy-*D*-mannoic lactone, and 2-fluoro-5-hydroxy-1-ribofuranosyl compounds, which have been associated with antioxidant, anti-inflammatory, cytotoxic, and hepatoprotective activities [12]. In addition, qualitative phytochemical screening has shown that *F. trinervia* extracts contain carbohydrates, flavonoids, polyphenols, saponins, tannins, triterpenoids, and phenolic compounds [9,11]. Notably, oleanolic acid, a well-known triterpenoid with hepatoprotective, anti-inflammatory, and analgesic properties, has also been reported and may contribute significantly to the observed pharmacological effects [10].

Networked pharmacology enables the study of the pharmacological basis and mechanisms of action of bioactive plant compounds for therapeutic purposes, the exploration of multiple drug components, the identification of targets of action, and the elucidation of potential mechanisms of action in disease [13]. The use of gas chromatography–mass spectrometry (GC-MS) in network pharmacology has allowed the examination of differences in active compounds and potential targets between medicinal (flower buds) and non-medicinal (flowers) raw materials [14].

Consequently, this study aims to compare the phytochemical profiles of *F. trinervia* (Spreng.) C. Mohr of GC-MS to analyze the potential pharmacological mechanisms of their compounds via network pharmacology based on network pharmacology and investigated the potential pharmacological basis and mechanism through the screening of active components and their targets in the database, the construction of ingredient–target–disease network, protein–protein interaction network, Gene Ontology (GO) enrichment analysis, Kyoto Encyclopedia of Genes and Genomes (KEGG) enrichment analysis, to identify bioactive compounds and relevant molecular pathways with particular emphasis on nuclear receptor signaling, metabolic regulation, immune response, and cancer-related processes.

## 2. Materials and Methods

### 2.1. Plant Collection and Identification

Fresh leaves of *F. trinervia* were collected in August 2021 from its natural habitat in the locality of San Felipe del Agua, municipality of Oaxaca de Juárez, Oaxaca, México (17° 06′ 46″ N, 96° 45′ 41″ W; altitude: 1550 m). Leaves were harvested from multiple mature plants (n > 10) during the flowering stage, under dry and sunny weather conditions, as confirmed by the presence of fully developed capitula along branched stems. Collection was performed between 07:00 and 08:00 h to minimize plant stress and potential diurnal variation or metabolite degradation, including phenolics, terpenoids, alkaloids, and other nitrogen-containing compounds (steroidal glycoalkaloids and glucosinolates) [15,16].

The plant material was taxonomically authenticated at the herbarium of the “Centro Interdisciplinario de Investigación para el Desarrollo Integral Regional (CIIDIR)”, Unidad Oaxaca, Mexico. All specimens were identified as *Flaveria trinervia* (Spreng.) C. Mohr (family Asteraceae) [17,18] and confirmed to be not listed under protection by the Mexican Official Standard NOM-059-SEMARNAT-2010. One representative specimen corresponds to the reproductive (flowering, see Supplementary Materials: phenological stage) stage, as evidenced by the presence of fully developed capitula, and was deposited in the Herbario OAX scientific collection under the voucher number OAX 35558 (see Supplementary Materials: taxonomic identification), while the remaining specimens will be distributed among other national herbaria for long-term preservation and reference. As *F. trinervia* is a non-model species, its genetic information is not available in a public genomic database. However, the botanical identification and voucher specimen are publicly accessible through the herbarium record mentioned above.

### 2.2. Extracts

The extractions were conducted at the Laboratorio de Biotecnología de la Facultad de Agronomía y Veterinaria, Universidad Autónoma de San Luis Potosí (UASLP), and at the Centro Regional de Biociencias, UASLP. We used 10 g (g) of leaves, which were macerated in 25 mL of chloroform (CHCl_3_, Sigma-Aldrich, St. Louis, MO, USA, EE.UU.). The mixture was vortexed for 1 h to prevent agglomeration and sedimentation. The extracts were filtered three times using Whatman filter paper, grades 1, 5, and 6 in sequence, under vacuum. Sample preparation for GC–MS analysis was performed by diluting the extract in acetone/CHCl_3_ ((CH_3_)_2_CO/CHCl_3_, Sigma-Aldrich, EE.UU.) and hexane/dichloromethane (C_6_H_14_/CH_2_Cl_2_, Sigma-Aldrich, EE.UU.) at a 1:1 ratio. These solvent systems were selected to ensure GC–MS compatibility and optimize injection and chromatographic separation by improving extract viscosity, compound solubility, and the handling of poorly volatile extracts, which can compromise peak shape, column efficiency, and detector response. compromising chromatographic system compatibility [19,20]. After extracts of (CH_3_)_2_CO/CHCl_3_ and C_6_H_14_/CH_2_Cl_2_ were filtered through polytetrafluoroethylene (PTFE, Sigma-Aldrich, EE.UU.) or polyvinylidene (PVDF, Sigma-Aldrich, EE.UU.) membranes (with different hydrophobic adsorption ranges and size exclusion pores) and then transferred to a vial for analysis by GC-MC [21].

### 2.3. Gas Chromatography Coupled to Mass Spectrometric (GC-MS) Analysis Scan Mode

This process was conducted at the Coordinación para la Innovación y la Aplicación de la Ciencia y la Tecnología, UASLP. We used the Hewlett-Packard gas chromatograph HP 6890, coupled to a mass spectrometry (MS) detector with an electronic impact HP 5973 (Agilent Technologies, Palo Alto, CA, USA). The column exerted was an absorbed silica capillary column of 95% methyl-poly-siloxane and 5% phenyl (HP-5MS; length: 60 m, diameter: 0.25 mm, and film 0.25 µm). Helium was used as carrier gas, and the flow rate was 20 mL/min. The GC oven temperature gradient was as follows: initially, 60 °C (hold for 3 min), then increased by 5 °C every minute until 300 °C, at which point it was held for 5 min. The transfer line temperature was 250 °C, and the pressure was 17.71 psi. The ion source temperature was 230 °C, and the mass spectrometer (MS) was scanned over the mass range 50–550 m/z. The assays were processed in the Agilent ChemStations software version 4.02 (Houston, TX, USA) to generate chromatograms for the interpretation of the spectra.

### 2.4. Identification of Phytochemical Compounds

Component identification was based on retention indices, and mass spectral interpretation was performed using the National Institute of Standards and Technology (NIST) database. The database contains over 62,000 patterns of known compounds. Compounds were tentatively identified by comparing their spectral fragmentation patterns with the standard reference spectrum using the NIST98 and WILEY275 libraries. Linear retention indices (LRIs) were not used in this study because their calculation requires the parallel analysis of certified n-alkane mixtures under chromatographic conditions identical to those of the samples [22,23], a procedure not included in the experimental design. Furthermore, the libraries available in the software used do not include an integrated database of retention indices, which limits direct comparison.

The area of the GC peaks depends not only on the concentration of the related compounds but also on the intensity of their spectral fragmentation. Nomenclature for all compound names was standardized with International Union of Pure and Applied Chemistry (IUPAC) rules. Relative quantification of the compounds present in each sample was obtained from the relative area of the peaks in the chromatograms. Consequently, the reported compounds should be considered presumptive identifications based on spectral agreement with reference libraries, rather than unequivocal identifications or confirmed by certified analytical standards. Reconstructed GC–MS chromatograms were generated using OpenChrom software (version 1.6.1), only peaks with a mass spectral similarity ≥80% were retained, numbered, and tentatively identified based on NIST library matching, while signals attributed to column bleed, instrumental artifacts, or contaminants were excluded. Biological activity for each identified compound was obtained with an exhaustive search in scientific publications.

### 2.5. Network Pharmacological Analysis

Potential active compounds were selected from the identified compounds using GC-MS: (a) (CH_3_)_2_CO/CHCl_3_, and (b) C_6_H_14_/CH_2_Cl_2_; and their molecular structures were confirmed via PubChem (https://pubchem.ncbi.nlm.nih.gov/) (20 January 2026) [24]. Only compounds with a similarity ≥80% and biological relevance were included in subsequent analyses, while compounds associated with analytical impurities or laboratory contaminants were excluded [25,26].

Analytical platforms and pharmacological databases in traditional medicine were employed (22 January 2026), these include to Swiss Target Prediction (http://www.swisstargetprediction.ch/), HERB (http://herb.ac.cn/), Indian Medicinal Plants, Phytochemistry, and Therapeutics (IMPPAT, https://cb.imsc.res.in/imppat/), and STITCH (http://stitch.embl.de/).

For each compound identified by GC-MS in both extracts, a systematic search was conducted in the selected platforms. Metabolites from the (CH_3_)_2_CO/CHCl_3_ extract were labeled with AC, while those from the C_6_H_14_/CH_2_Cl_2_ extract were coded with the prefix HD, to ensure traceability throughout the analysis. Target prediction was restricted to Homo sapiens, and duplicate targets within each extract were excluded to avoid redundancies.

Based on this classification, three molecular target datasets were constructed: targets from the HD extract, targets from the AC extract, and shared targets between both extracts. Using these datasets, three interaction networks were generated in Cytoscape (v.3.10.3): (i) the HD extract network, (ii) the AC extract network, and (iii) the common targets network. The topological properties of each network were evaluated using the CytoHubba plugin version 0.1 [27], with a focus on the most relevant parameters for identifying key nodes: degree, betweenness centrality, closeness centrality, and clustering coefficient.

### 2.6. Construction and Analysis of Protein–Protein Interaction Networks

The STRING database (https://string-db.org/, 23 January 2026) was used to evaluate protein–protein interactions (PPIs) from three datasets derived from *Flaveria trinervia* (Spreng.). C. Mohr extracts and the shared targets. This platform integrates information on protein–gene interactions based on validated experimental data, computational predictions, and publicly available literature. The protein targets identified in each extract and in the shared set were uploaded to STRING, with *Homo sapiens* selected as the reference species. For subsequent analysis, PPIs were filtered to retain only those with a high confidence score (≥1) [28].

### 2.7. Gene Ontology Functional Enrichment and Pathway Analysis

From a systemic perspective, target proteins tend to interact with multiple biological processes and cellular components rather than functioning independently. In this study, the targets obtained from the PPIs network analysis of the common extract-derived targets were introduced into STRING, an online tool, to perform Gene Ontology (GO) functional enrichment and pathway analysis. STRING is a web-based suite of gene set analysis tools. Enrichment analyses were conducted for biological processes, molecular functions, cellular components, gene–disease associations, tissue expression, and KEGG pathways across all genes in the network, using an FDR < 0.05 as the significance threshold.

### 2.8. Construction of the Active Component–Target–Disease Network

Diseases were identified using DisGeNEt (http://www.disgenet.org/, 23 January 2026) based on targets with evidence of satisfactory clinical trial outcomes [29]. These targets were selected from the top 20 results of the PPIs network analysis of the compounds common to both extracts. Subsequently, the active component–target–disease network was constructed in Cytoscape (v. 3.10.3).

## 3. Results

### 3.1. Identification of Extract Composition

**Figure 1:**
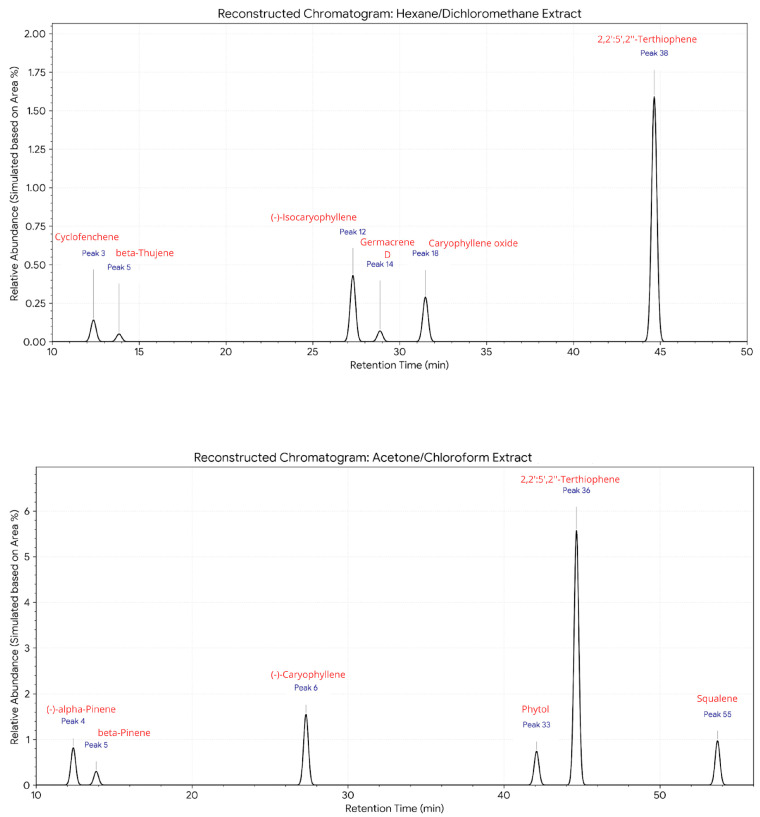
Reconstructed GC–MS chromatograms of the extract of *F. trinervia* leaves. The peaks corresponding to compounds with a mass spectral similarity ≥80%.

The GC-MS analysis of *F. trinervia* leaf extracts identified 68 compounds in the hexane/dichloromethane mixture and 61 compounds in the acetone/chloroform mixture. Reconstructed GC–MS chromatograms of *Flaveria trinervia* leaf extracts obtained using (A) hexane/dichloromethane and (B) acetone/chloroform solvent systems. Only peaks corresponding to compounds with a mass spectral similarity ≥80% are shown, Figure 1. Each peak is numbered and labeled with the tentatively identified compound name based on mass spectral matching with the NIST library. Peaks attributed to column bleed, instrumental artifacts, or other contaminants were excluded.

The detailed information on the identified compounds, including retention time (RT), compound name, CAS number, and similarity (%), is provided in Table 1 and Table 2. It is important to note that the compounds listed in Table 1 and Table 2 correspond only to those metabolites whose spectral matches to the reference libraries were ≥80% and have documented biological relevance. Peaks attributable to column bleed, instrumental contaminants, or analytical artifacts were excluded from the analysis and the final tables to avoid misinterpretations and ensure the reliability of subsequent analyses. In the hexane/dichloromethane mixture (Table 1), the following bioactive compounds were tentatively identified: Cyclofenchene, beta-Thujene, (-)-Isocaryophyllene, Germacrene D, Caryophyllene oxide, and 2,2′:5′,2″-Terthiophene. In that, the acetone/chloroform mixture (Table 2), the bioactive compounds detected included: (+/-)-alpha-Pinene, Beta-Pinene, (-)-Caryophyllene, Phytol, 2,2′:5′,2″-Terthiophene, and Squalene. 

### 3.2. Identification of Targets from Extract Composition

A total of 448 potential targets were identified for the hexane/dichloromethane extract, 588 potential targets for the acetone/chloroform extract, and 197 common targets for the compounds shared by both extracts, based on searches restricted to *Homo sapiens* in the Swiss Target Prediction, HERB, Indian Medicinal Plants, IMPPAT, and STITCH databases.

**Figure 2:**
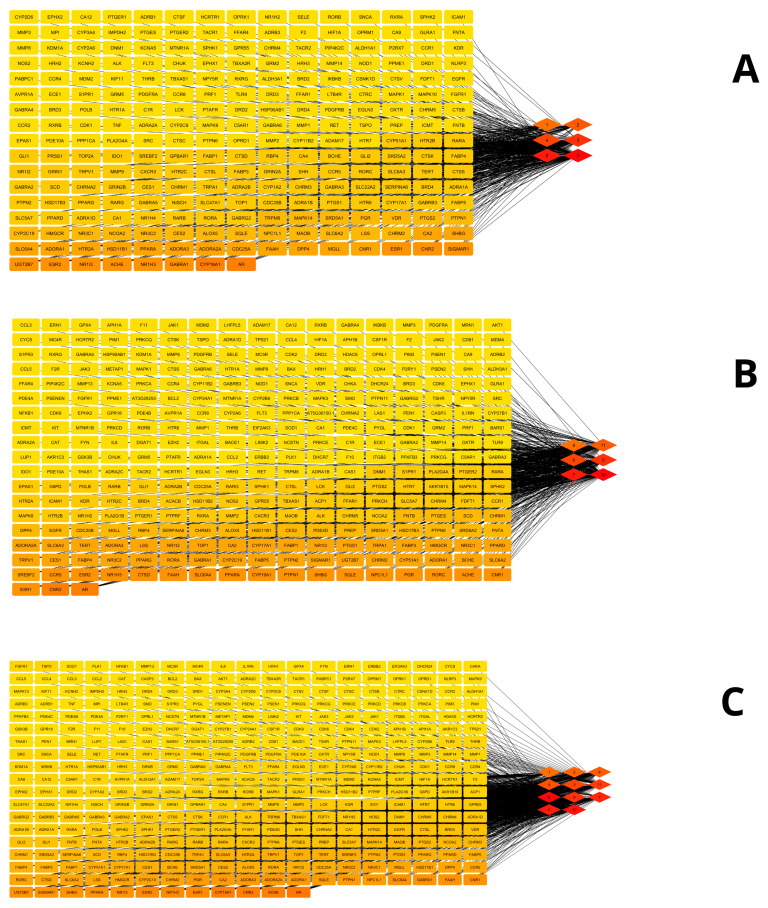
Network of components and targets from the composition of *F. trinervia* (Spreng.) C. Mohr leaf extracts: hexane/dichloromethane extract (**A**), acetone/chloroform extract (**B**), and common compounds (**C**). The color scale, ranging from red to yellow, illustrates the degree of interaction in descending order.

After constructing the component–target interaction networks, 248 nodes were visualized for the hexane/dichloromethane extract (Figure 2A), 254 nodes for the acetone/chloroform extract (Figure 2B), and 315 nodes for the common compounds (Figure 2C). In these networks, circular nodes represent protein targets, while diamond-shaped nodes correspond to the chemical components of the extracts. 

### 3.3. Construction and Topological Analysis of Protein–Protein Interaction Networks

**Figure 3:**
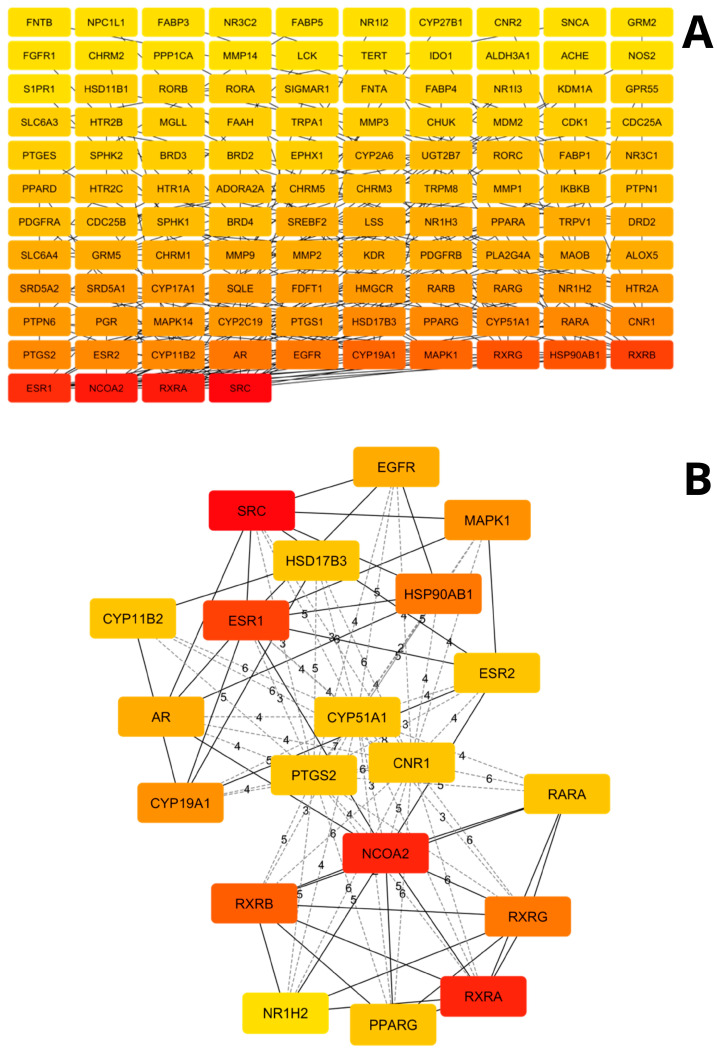
Protein–protein interaction (PPI) network of the common compounds identified in *F. trinervia* (Spreng.) C. Mohr leaf extracts. (**A**) Complete interaction network. (**B**) Subnetwork of the top 20 ranked nodes. The color scale, ranging from red to yellow, illustrates the degree of interaction in descending order.

The PPIs network constructed from all common compounds of the extracts is shown in Figure 3A, while a visualization of the top 20 nodes is presented in Figure 3B to enhance interpretability. The complete network comprises 114 nodes, representing the targets, and 214 edges, corresponding to associations between pairs of targets.

Topological analysis (Table 3) revealed that SRC is the node with the highest number of interactions (16), followed by RXRA (14), NCOA2 (14), ESR1 (12), RXRB (11), HSP90AB1 (9), RXRG (9), MAPK1 (8) and EGFR (8).

### 3.4. GO Enrichment and Pathway Analysis

GO functional enrichment analysis provided a comprehensive characterization of the biological processes, molecular functions, and cellular components associated with target proteins derived from common compounds in *F. trinervia* extracts. Functional enrichment analysis of the identified target genes revealed a significant association with multiple biological processes and signaling pathways.

The enrichment of biological processes indicates a predominant involvement in nuclear receptor–mediated signal transduction, as evidenced by the overrepresentation of intracellular receptor signaling pathways, particularly those modulated by steroid hormones and retinoic acid receptors. This suggests that the bioactive components may act as agonist or antagonist ligands, capable of translocating to the nucleus and regulating gene expression by interacting with hormone response elements in DNA.

**Figure 4:**
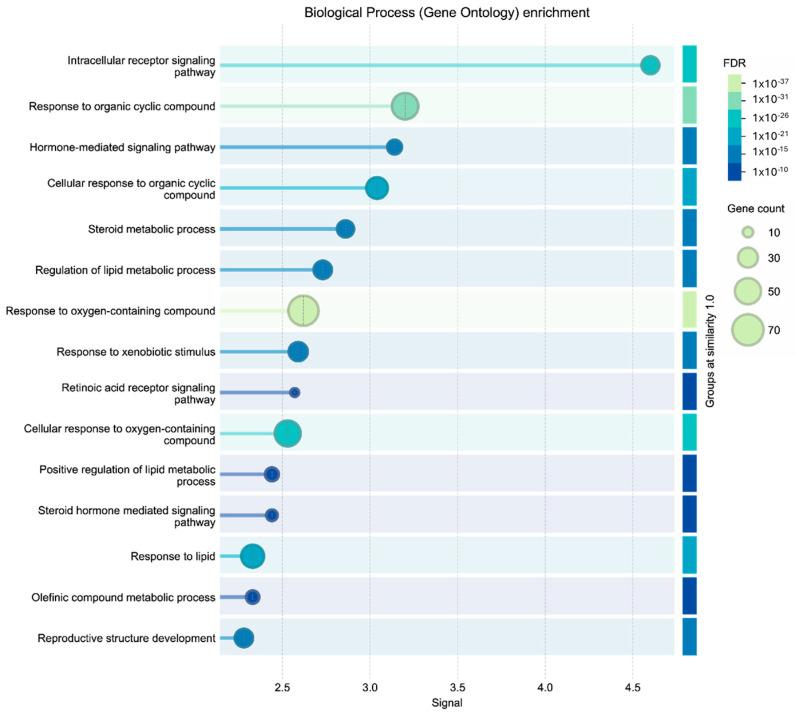
Biological process (gene ontology) enrichment.

In addition, a significant reprogramming of lipid and steroid homeostasis was observed, characterized by the upregulation of lipid metabolic processes and olefinic compound metabolism. This metabolic activity closely correlates with cellular responses to xenobiotic stimuli and cyclic organic compounds, implying the activation of biotransformation and detoxification mechanisms, likely involving phase I and phase II enzymes, as an adaptive strategy to chemical exposure. Such processes may ultimately influence complex physiological outcomes, including the development of reproductive structures (Figure 4). Specifically, the hierarchy of significance, dominated by key mechanisms such as nuclear receptor signaling, regulation of lipid/steroid metabolism, and response to xenobiotics, supports the hypothesis that the compounds identified by GC-MS do not act in isolation, but exert their biological effects through a pleiotropic and multi-target mechanism, modulating integral networks of adaptation and cell signaling.

The enriched terms exhibited extremely low False Discovery Rate (FDR) values, indicating high statistical reliability. Furthermore, several processes showed a high number of associated genes, suggesting coordinated involvement of multiple molecular targets. In particular, the terms with the highest significance and gene counts were related to key cellular mechanisms, including regulation of cell proliferation, immune signaling, and stress response processes, suggesting that the compounds identified by GC-MS could exert biological effects through a multi-target mechanism.

**Figure 5:**
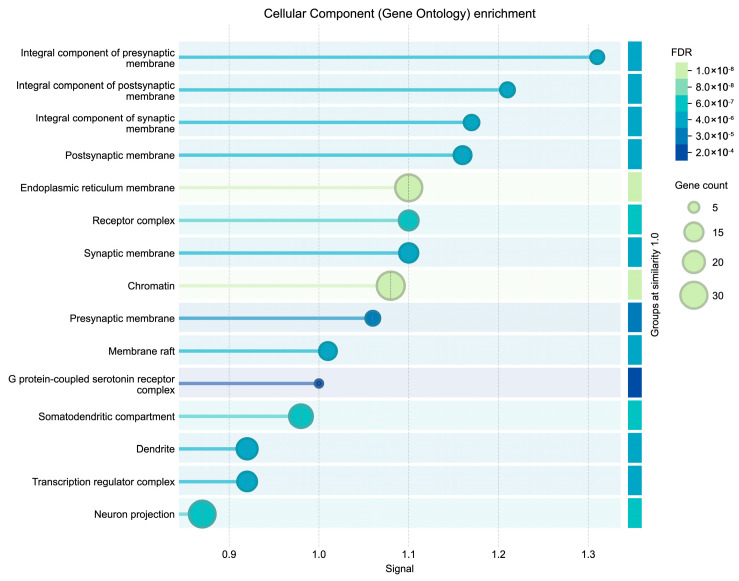
Cellular component (gene ontology) enrichment.

Gene ontology analysis for Cellular Components reveals highly specific subcellular compartmentalization, with a predominant localization in synaptic architecture. A significant enrichment of integral proteins was identified in pre- and postsynaptic membranes, as well as in the somatodendritic compartment and neuronal projections, suggesting a direct involvement of metabolites in neurotransmission machinery and the maintenance of synaptic plasticity. Furthermore, the localization in membrane rafts, lipid microdomains essential for signal transduction, and specifically in the serotonin receptor G protein-coupled complex, is noteworthy, pointing to modulation of serotonergic pathways. Finally, the identification of nuclear components such as chromatin and the transcription regulatory complex reinforces the hypothesis of a dual mechanism of action: rapid membrane signaling and long-term genomic regulation (Figure 5).

**Figure 6:**
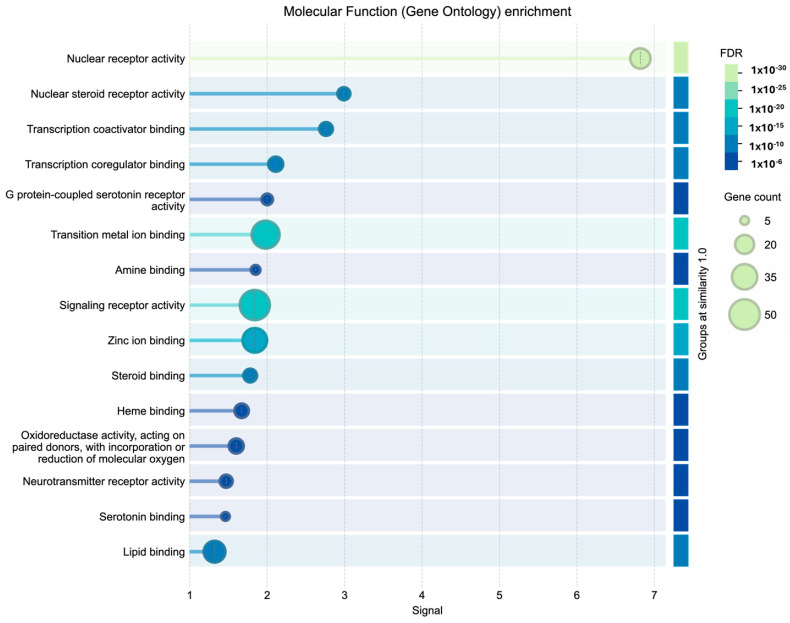
Molecular function (gene ontology) enrichment.

Regarding molecular function, an enrichment was observed in nuclear receptor activity, transcription corepressor binding, and monooxygenase activity, indicating possible roles in transcriptional regulation and in oxidation-reduction processes essential for maintaining cellular homeostasis (Figure 6).

Regarding molecular function, the enrichment profile reveals a dual function. On the one hand, nuclear receptor activity (specifically steroid receptors) predominates, coupled with the binding of transcription coactivators and co-regulators, confirming the compounds’ ability to modulate gene expression machinery through macromolecular complexes. On the other hand, significant activity linked to neurochemical signaling emerges, notably the activity of G protein-coupled serotonin receptors and binding to amines and neurotransmitters.

**Figure 7:**
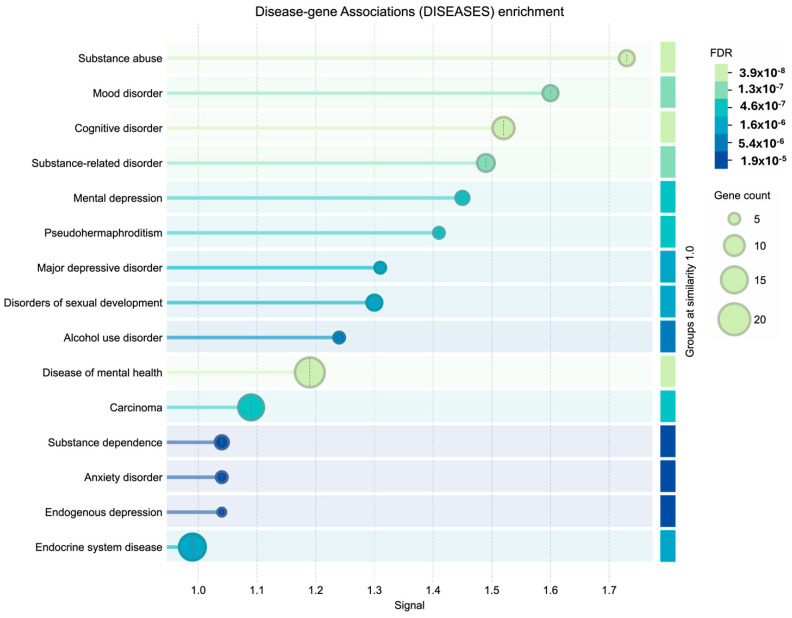
Disease-gene association (diseases) enrichment.

Additionally, the prominence of binding to transition-metal ions (mainly zinc) and heme, along with oxidoreductase activity, suggests the critical role of metal cofactors in both the structural integrity of transcription factors (zinc finger motifs) and redox enzyme catalysis (Figure 7). The results show significant enrichment in biological processes related to nuclear receptor-mediated signaling and to the response to xenobiotic and cyclic organic compounds. A strong representation of terms associated with lipid and steroid metabolism is also observed, suggesting that common *F. trinervia* metabolites could modulate metabolic and cellular detoxification pathways. Overall, the enrichment pattern indicates that the identified bioactive compounds act in a multi-target manner, converging on processes of transcriptional regulation, metabolism, and adaptive response to chemical stimuli. This is consistent with the network analysis results and supports their potential relevance in inflammatory and tumor contexts.

**Figure 8:**
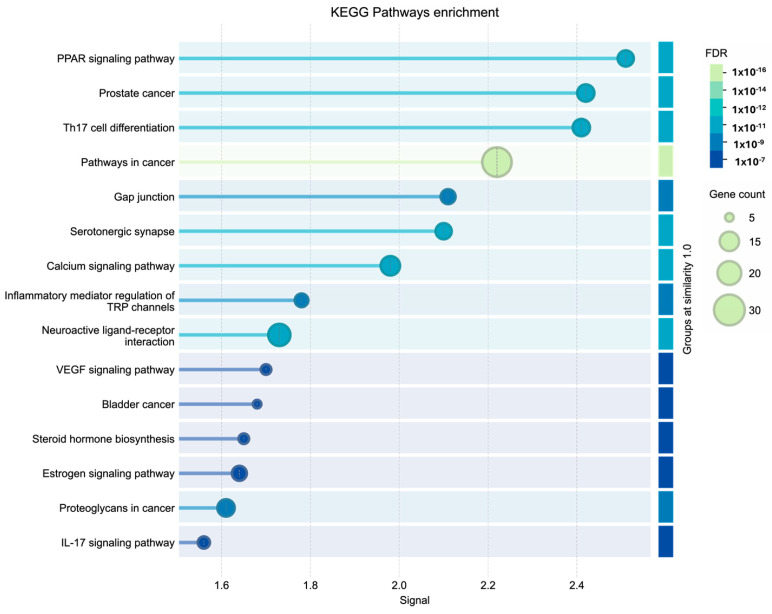
KEGG pathway enrichment analysis of the potential targets.

Finally, the KEGG pathway enrichment analysis revealed a complex, multifaceted biological interaction network (Figure 8). Significant prominence was observed in pathways related to molecular oncology, including “Pathways in cancer” and “Prostate cancer”, suggesting a potential influence on the signaling mechanisms governing abnormal cell proliferation and survival. Crucially, the analysis highlighted a strong immunomodulatory and inflammatory response, evidenced by enrichment in Th17 cell differentiation and the IL-17 signaling pathway. Furthermore, high-level metabolic and nuclear signaling mechanisms were identified, including the PPAR signaling pathway, steroid hormone biosynthesis, and estrogen signaling. Concurrently, involvement in neuronal signal transduction was observed, including serotonergic synapses, calcium signaling, and neuroactive ligand-receptor interactions.

### 3.5. Construction of the Active Component–Target–Disease Network

The diseases identified in the DISGENET database were classified into the following categories: Tumors, Immune, Metabolic and Endocrine, Neurological and Psychiatric, and Cardiovascular. The analysis of compound-disease associations revealed a broad spectrum of potential therapeutic targets. In the context of solid tumors, it was observed that major compounds such as Germacrene D, Caryophyllene, and Pinene isomers share molecular targets (ESR1, CYP19A1, NCOA2) linked to the progression of breast, prostate, endometrial, and ovarian cancers (Table 4). Likewise, specific interactions relevant to aggressive pathologies were identified: Squalene and Phytol were associated with the blockade of EGFR signaling in non-small cell lung cancer (NSCLC) and glioblastoma, while Caryophyllene Oxide was linked to the regulation of HIF1A, a crucial factor in the hypoxic tumor microenvironment. Finally, the search yielded evidence of activity against hematological neoplasms (myeloid leukemia and lymphomas), mediated mainly by modulation of retinoid receptors (RXR) and heat shock protein 90 (HSP90AB1).

In the category of immune disorders (Table 5), compound–target association analysis revealed significant links with pathologies of autoimmune and viral etiology. Specifically, terpenes such as Germacrene D and (+/-)-alpha-Pinene were found to be associated with the potential treatment of Systemic Lupus Erythematosus and Granulomatosis with Polyangiitis by modulating the nuclear receptor RXRB. Additionally, relevant associations were identified for chronic inflammatory diseases such as Multiple Sclerosis and Asthma, as well as for HIV infections. These pathologies share the HSP90AB1 protein as a molecular target, which is modulated by compounds present in the extract, such as Squalene and 2,2′:5′,2″-Terthiophene.

In categorizing metabolic and endocrine diseases (Table 6), data mining enabled the establishment of a preliminary molecular link between the identified metabolites and various chronic pathologies. The search revealed that major compounds such as beta-pinene, caryophyllene, squalene, and cyclofenchene have been shown to interact with critical hormonal regulators, specifically Estrogen Receptor 1 (ESR1) and Aromatase (CYP19A1). These molecular targets support the association of the extracts with disorders of the reproductive and skeletal axes, such as Polycystic Ovary Syndrome, Endometriosis, and Osteoporosis. In parallel, evidence was compiled linking Germacrene D, (+/-)-alpha-pinene, and Phytol to the regulation of energy homeostasis through the Retinoid X Receptor family (RXRA, RXRB, RXRG). These associations suggest a potential involvement in systemic lipid dysregulation, including obesity, metabolic syndrome, and dyslipidemia. Additionally, caryophyllene oxide was identified as a modulator of the HIF1A factor, establishing a specific molecular link with type 2 diabetes mellitus.

In the field of nervous system pathologies (Table 7), compound–target association analysis revealed a pleiotropic activity profile. The most predominant association was with Alzheimer’s disease, linked to most of the terpenes and diterpenes present (e.g., pinene isomers, caryophyllene, phytol) through the modulation of key neuroendocrine targets: ESR1 and CYP19A1. On the other hand, a subgroup of compounds, led by germacrene D and (+/-)-alpha-pinene, was identified as potentially active against cognitive and movement disorders. These metabolites were associated with schizophrenia, Parkinson’s disease, memory disorders, and intellectual disability, mediated by the regulation of transcription factors (RXRB) and kinase signaling pathways (MAPK1). Additionally, a connection was observed with proteotoxic stress response mechanisms in general neurodegeneration (HSP90AB1), mediated by squalene.

In cardiovascular health (Table 8), compound–target association analysis revealed significant interactions with both congenital and acquired pathologies. A prominent finding was the link between major terpenes (Germacrene D, alpha-Pinene, Phytol) and Congenital Heart Defects and Atherosclerosis, mediated by interaction with Retinoid Receptor X isoforms (RXRA, RXRB, RXRG), key regulators of cell differentiation and lipid metabolism. Also, mechanisms related to ischemic and hypertensive pathology were identified. Specifically, caryophyllene oxide was associated with myocardial infarction through the hypoxia signaling pathway (HIF1A). Likewise, Squalene and Phytol showed potential in the management of Hypertension, associating with targets involved in vascular growth and stress such as EGFR and the heat shock protein HSP90AB1.

**Figure 9:**
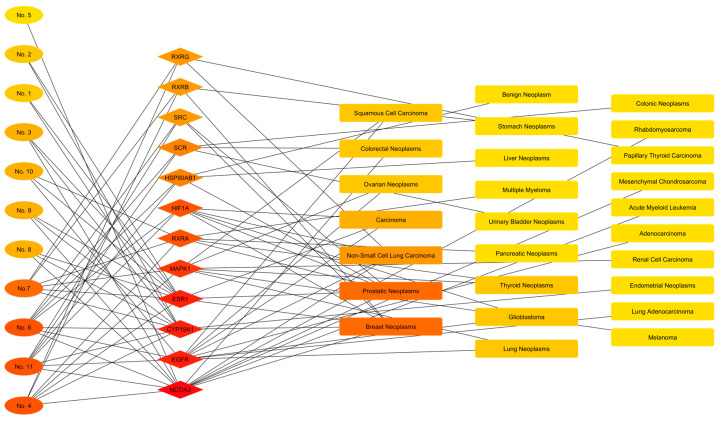
Active component–target–tumor network of *Flaveria trinervia* leaf extracts. The color scale, ranging from red to yellow, illustrates the degree of interaction in descending order.

**Figure 10:**
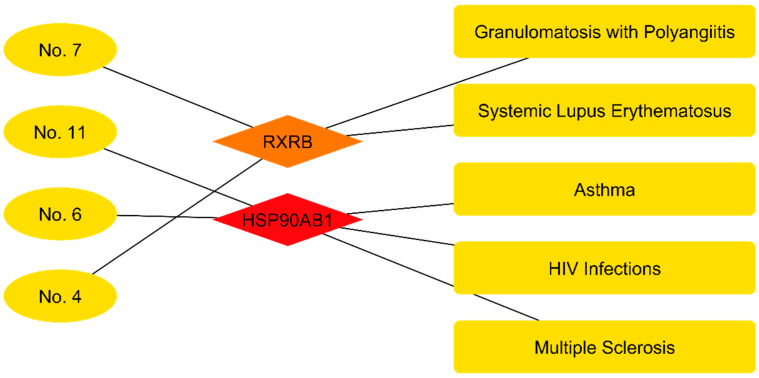
Compound–target–disease network highlighting immune and inflammatory disorders modulated by *Flaveria trinervia*. The color scale, ranging from red to yellow, illustrates the degree of interaction in descending order.

**Figure 11:**
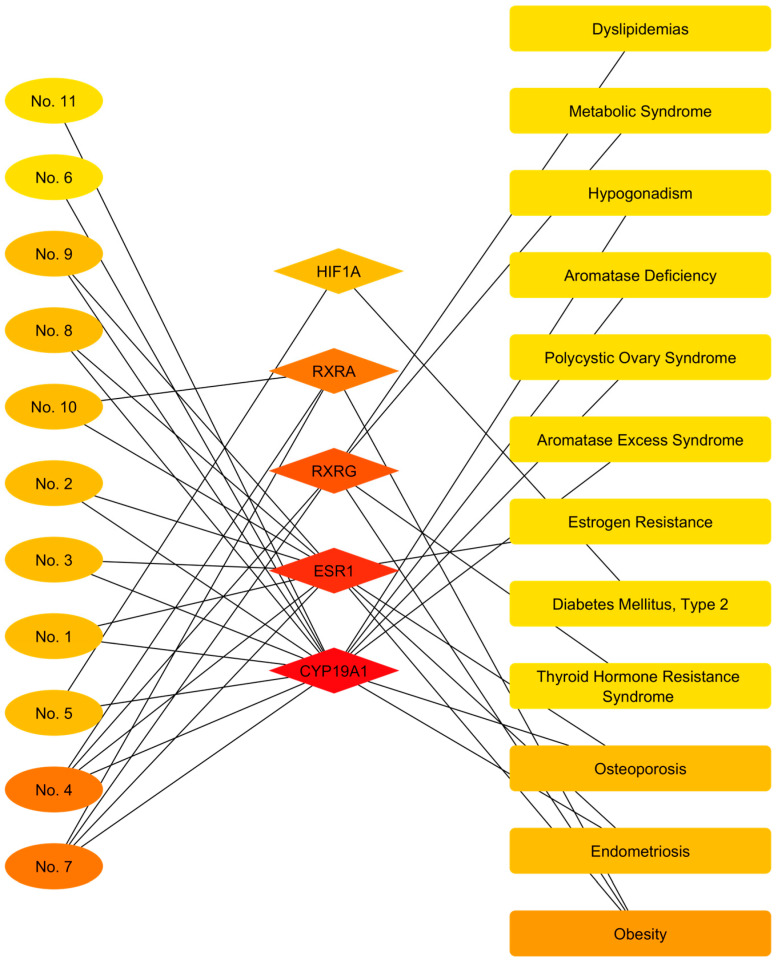
Active component–target–metabolic and endocrine disease network of *Flaveria trinervia*. The color scale, ranging from red to yellow, illustrates the degree of interaction in descending order.

**Figure 12:**
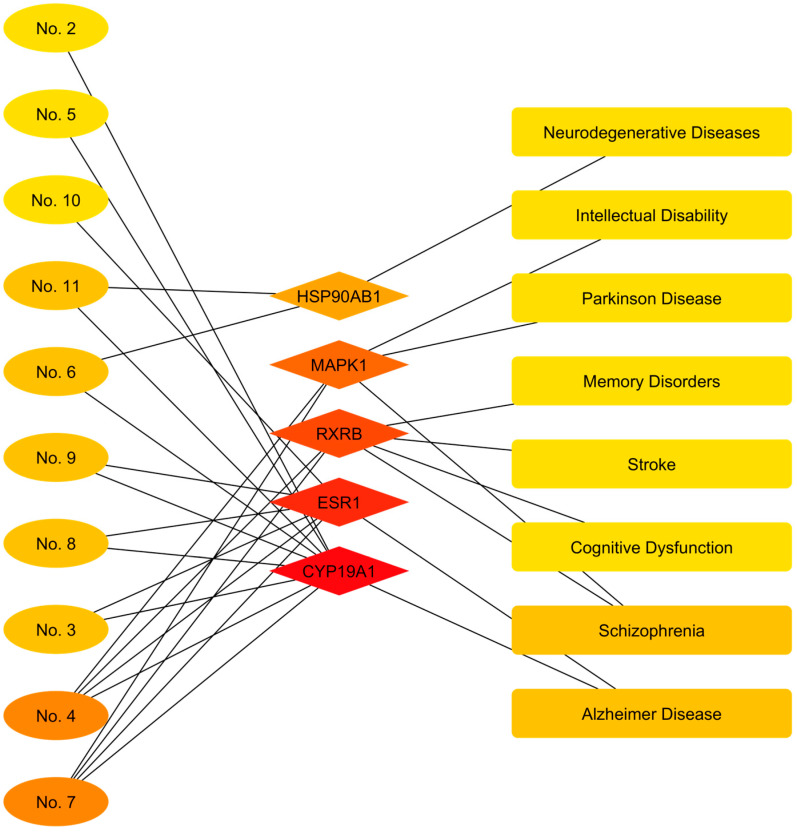
Network representation of bioactive compounds, molecular targets, and neurological disorders associated with *Flaveria trinervia*. The color scale, ranging from red to yellow, illustrates the degree of interaction in descending order.

**Figure 13:**
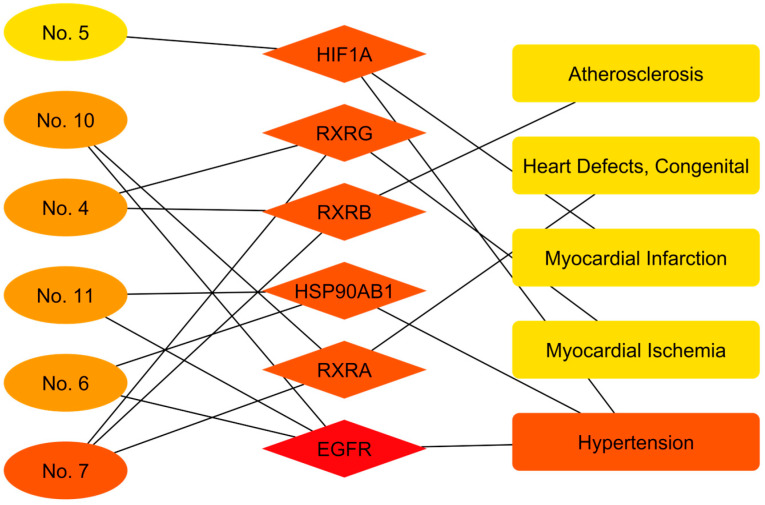
Compound–target–cardiovascular disorder interaction network of *Flaveria trinervia*. The color scale, ranging from red to yellow, illustrates the degree of interaction in descending order.

The Active Component-Target-Tumor networks were constructed (Figure 9, Figure 10, Figure 11, Figure 12 and Figure 13). Topological analysis reveals that the interaction is not linear but rather organized through highly connected central nodes (‘hubs’), represented by the larger, more intensely colored rhombuses.

The construction of the Compound–Target–Tumor interactive network revealed a complex topology, characterized by multiple highly connected molecular targets that articulate the interactions between metabolites and pathological phenotypes. Centrality analysis identified three main interaction modules in the target layer (Figure 9). The first module, defined by the highest edge density, was organized around Estrogen Receptor 1 (ESR1), aromatase (CYP19A1), and Nuclear Receptor Coactivator 2 (NCOA2). These nodes acted as convergence points for most of the analyzed compounds (Nos. 1–4, 7–10) and projected their connections primarily toward hormone-dependent disease nodes, specifically breast, prostate, and endometrial neoplasms. In parallel, a second cluster was identified, structured around the Epidermal Growth Factor Receptor (EGFR) and the MAPK1 kinase. This module exhibited specific connections originating from the compounds Squalene (No. 11) and Phytol (No. 10), directly linking them to non-small cell lung carcinoma and glioblastoma. Finally, a third functional cluster was observed involving the Retinoid X Receptor family (RXRA, RXRB, and RXRG), which predominantly linked the compounds Germacrene D (No. 4) and (+/-)-alpha-Pinene (No. 7) with thyroid and hematological neoplasms, such as acute myeloid leukemia.

Analysis of the Compound–Target–Immune Disorder network (Figure 10) revealed a segmented topology in two independent clusters. The first cluster showed the Retinoid X Receptor Beta (RXRB) as the central node, receiving edges from the compounds Germacrene D (No. 4) and (+/-)-alpha-Pinene (No. 7). Outgoing connections from this node were directed exclusively toward systemic autoimmune diseases: Systemic Lupus Erythematosus and Granulomatosis with Polyangiitis. The second cluster was organized around the HSP90AB1 protein, which exhibited interactions with 2,2′:5′,2″-Terthiophene (No. 6) and Squalene (No. 11). In this module, the pathological associations were heterogeneous, linking this molecular target to asthma, multiple sclerosis, and HIV infections. Unlike the tumor network, no shared nodes were observed between these two functional modules.

Topological analysis of the network, focused on metabolic and endocrine disorders (Figure 11), revealed a hierarchical modular architecture, dominated by three independent signaling axes that distribute metabolite interactions to specific pathological groups. The module with the highest density and centrality was structured around Estrogen Receptor 1 (ESR1) and the aromatase enzyme (CYP19A1). These nodes acted as the main receptors for edges originating from the vast majority of the identified compounds (No. 1–3, 8–11), channeling these interactions toward a cluster of diseases associated with dysregulation of the reproductive axis and bone density, including polycystic ovary syndrome, endometriosis, aromatase excess/deficiency syndromes, and osteoporosis. Simultaneously, a second functional module governed by the Retinoid Receptor X isoforms (RXRA and RXRG) was identified. Unlike the estrogen axis, these nodes exhibited selective connectivity with the compounds Germacrene D (No. 4) and (+/-)-alpha-Pinene (No. 7). Outgoing connections from this cluster of nuclear receptors were directly linked to alterations in systemic and lipid metabolism, including obesity, metabolic syndrome, dyslipidemia, and thyroid hormone resistance syndrome. Finally, the network highlighted an isolated signaling pathway mediated by Hypoxia-Inducible Factor 1-alpha (HIF1A). This node established a unique and exclusive link between Caryophyllene Oxide (No. 5) and Type 2 Diabetes Mellitus, without overlapping with the previously described hormonal or lipid modules.

Visualization of the network focused on neurological and psychiatric disorders (Figure 12) revealed a functional architecture clearly segmented into three interaction modules, defined by the specificity of the molecular targets involved. The predominant module, characterized by the highest density of incoming connections, was organized around Estrogen Receptor 1 (ESR1) and aromatase (CYP19A1). These central nodes acted as primary receptors for the vast majority of the extract’s compounds (including nodes 2–5 and 7–10), converging unequivocally toward a single output node: Alzheimer’s disease. This topological configuration demonstrates that the neuroendocrine pathway serves as the primary association mechanism linking the major metabolites to this neurodegenerative pathology.

Simultaneously, a second signaling axis was identified, articulated by Retinoid Beta X Receptor (RXRB) and the MAPK1 kinase. This cluster exhibited marked selectivity, receiving edges exclusively from the compounds Germacrene D (No. 4) and (+/-)-alpha-Pinene (No. 7). Projections from these intermediate nodes extended across a broad spectrum of cognitive and motor disorders, establishing direct links with Schizophrenia, Parkinson’s Disease, Stroke, Memory Disorders, and Cognitive Dysfunction. Finally, an independent module mediated by the heat shock protein HSP90AB1 was observed, which connected the compounds Squalene (No. 11) and 2,2′:5′,2″-Terthiophene (No. 6) to the general category of Neurodegenerative Diseases, also sharing a secondary connection with Alzheimer’s Disease.

The topological analysis of the cardiovascular pathology-oriented network (Figure 13) delineated a structure organized into three distinct functional modules, defined by the specificity of the interactions between the metabolites and their molecular targets. The first module, characterized by high connectivity, was structured around the Retinoid X Receptor family (RXRA, RXRB, and RXRG). These nodes acted as receptor hubs for the compounds Germacrene D (No. 4), (+/-)-alpha-Pinene (No. 7), and Phytol (No. 10), channeling their interactions toward pathologies related to vascular development and perfusion, specifically Congenital Heart Defects, Atherosclerosis, and Myocardial Ischemia. The second interaction module was defined by the convergence of the compounds Squalene (No. 11), 2,2′:5′,2″-Terthiophene (No. 6), and Phytol (No. 10) on the HSP90AB1 and Epidermal Growth Factor Receptor (EGFR) targets. The prominent edges of this cluster were unequivocally linked to hypertension, demonstrating a distinct signaling pathway that regulates vascular tone and remodeling. Finally, an independent axis mediated by Hypoxia-Inducible Factor 1-alpha (HIF1A) was observed, establishing a unique connection between Caryophyllene Oxide (No. 5) and myocardial infarction, suggesting a mechanism of action linked to the tissue response to acute hypoxic stress.

## 4. Discussion

In this study, we combined GC–MS analysis and network pharmacology to characterize the phytochemical profile of *F. trinervia* leaf extracts and to predict the potential multi-target and multi-pathway biological effects of its metabolites. The dual extraction approach (hexane/dichloromethane vs. acetone/chloroform) revealed a rich composition of terpenoids, siloxanes, and thiophene derivatives, with 11 bioactive compounds showing relevant similarity scores and seven compounds shared between both extracts, including cyclofenchene, β-thujene, (-)-isocaryophyllene, germacrene D, caryophyllene oxide, 2,2′:5′,2″-terthiophene, (+/-)-α-pinene, β-pinene, (-)-caryophyllene, phytol, and squalene.

These findings are broadly consistent with earlier phytochemical reports on *F. trinervia*. Ethanolic and methanolic extracts of the plant have previously been shown to contain flavonoids, terpenoids, and sulfur-containing compounds, along with antioxidant and cytotoxic properties in vitro [12]. GC–MS–based profiling of ethanolic extracts similarly identified multiple terpenes and fatty-acid derivatives, supporting the view that *F. trinervia* is a chemically diverse species with pharmacologically active constituents [12]. Our work extends these observations by comparing two organic solvent systems and by focusing on a subset of compounds whose predicted targets cluster in cancer- and immunity-related pathways.

Several of the key metabolites identified here have independent experimental support for biological activities that align with our network predictions. Phytol, detected in both extracts, has been widely reported as an antioxidant, anti-inflammatory, and cytotoxic compound that induces apoptosis and autophagy in various cancer cell lines, including lung cancer models [30,31,32]. Reports indicate that phytol exhibits lung anticancer activity by inhibiting the PI3K-Akt signaling pathway and may therefore be applicable for prevention and clinical treatment [33]. Terthiophene derivatives, particularly those from *Asteraceae*, have demonstrated antimicrobial, antifungal, and notable antitumor activities, including cytotoxic effects on tumor cells and antiproliferative actions in protozoal and fungal systems [34,35]. These reports lend biological plausibility to our prediction that 2,2′:5′,2″-terthiophene may contribute to anti-tumor effects by targeting SRC and EGFR.

Additionally, 2,2′:5′,2″-terthiophene and squalene were predicted to be associated with multiple cancer types through their interactions with key molecular targets, particularly SRC, CYP19A1, HSP90AB1, and EGFR. Network analysis revealed that these targets link both compounds to a broad spectrum of neoplastic conditions, including breast, colorectal, and colonic neoplasms; urinary bladder and liver neoplasms; endometrial and ovarian neoplasms; and lung-related malignancies, such as non-small cell lung carcinoma, lung adenocarcinoma, and lung neoplasms. Other associations were observed with glioblastoma, squamous cell carcinoma, adenocarcinoma, carcinoma, and benign neoplasms. In this regard, there is evidence that squalene epoxidase (SQLE) induces an impaired antitumor response in metabolic dysfunction-associated steatohepatitis-associated hepatocellular carcinoma (MASH-HCC) by attenuating CD8 T cell function and increasing myeloid-derived suppressor cells (MDSCs) [36]. Therefore, SQLE is a promising target for enhancing anti-PD-1 immunotherapy in MASH-HCC.

Similarly, sesquiterpenes such as germacrene D and caryophyllene oxide exhibit anti-inflammatory, antioxidant, and cytotoxic activities across various experimental models, including cancer [37]. In our analysis, germacrene D showed associations with MAPK1, TNF, and RXRA. At the same time, caryophyllene oxide was linked to CYP3A4, suggesting potential involvement in MAPK and nuclear receptor signaling, xenobiotic metabolism, and inflammatory modulation. These targets intersect with pathways commonly dysregulated in solid tumors and hematological malignancies, consistent with tumor indications identified in DISGENET.

The network pharmacology results strengthen the hypothesis that *F. trinervia* acts through a multi-target, multi-pathway mechanism. Topological analysis of the PPI network highlighted SRC, ESR1, RXRA/B/G, NCOA2, and CYP19A1 as central nodes with high degree values. These are core regulators of apoptosis, genomic stability, proliferation, and survival, and their dysregulation is a hallmark of many cancers. It is worth mentioning that ESR1, the gene spanning q24 to q27 of chromosome 6 and encoding the estrogen receptor alpha (ER), is the nuclear transcription factor most commonly implicated in breast cancer [38]. Evidence suggests that RXRs may exert direct beneficial effects in the heart through heterodimerization with other nuclear receptors (NRs) and homodimerization, making them suitable targets for treating cardiovascular diseases [39]. Enrichment in cancer-related KEGG pathways, including generic “pathways in cancer”, prostate cancer, and non-small cell lung cancer, is therefore consistent with both the predicted targets and the known activities of phytol, terthiophenes, and terpenoids reported in the literature [40,41,42].

Our findings also align with previous pharmacological reports in non-oncological models. *F. trinervia* leaf extracts have shown hepatoprotective effects in CCl_4_-induced liver injury, where they improved biochemical parameters and histology, suggesting robust antioxidant and membrane-stabilizing properties [9]. Additionally, they have described wound-healing, antioxidant, anti-inflammatory, and antipyretic activities [9]. Together with our gene-disease enrichment in immune disorders (rheumatoid arthritis, psoriasis, atopic eczema) and vascular diseases, these data support a convergent picture in which *F. trinervia* metabolites modulate inflammatory and immune pathways, partly via JAK1/JAK2 and TNF, as suggested by our networks.

Methodologically, the present work aligns with recent advances in network pharmacology applied to natural products and traditional medicines, integrating chemical profiling with system-level target prediction and pathway enrichment. This approach has been used, for example, to support quality control and mechanism-of-action studies in herbal preparations and essential oils [12,32,37]. Our study adds to this growing body of work by using *F. trinervia* as a model to explore how multiple leaf-derived compounds may converge on oncogenic and immunomodulatory networks.

Nevertheless, several limitations must be acknowledged. First, all interactions between compounds and targets were predicted in silico; experimental validation of binding, pathway modulation, and functional effects in relevant tumor and immune models remains necessary. Second, GC–MS primarily captures volatile and semi-volatile constituents, so non-volatile phenolics and flavonoids previously reported in *F. trinervia* may be underrepresented in our dataset [9]. Third, the disease associations derived from databases such as DISGENET reflect targets with existing clinical evidence, which may bias the network toward well-studied cancers and inflammatory diseases and underrepresent novel indications.

Given the large number of diseases associated with *Flaveria trinervia*, this suggests a multi-target action of the metabolites, potentially mediated by pathways related to estrogen biosynthesis and signaling, lipid metabolism, and endocrine homeostasis, consistent with the central molecular nodes identified in the interaction analysis.

From an analytical perspective, this study has some limitations that should be considered when interpreting the results. First, metabolite identification was based on a GC-MS screening approach designed to detect volatile and semi-volatile compounds. Therefore, non-volatile metabolites or those present in low concentrations may not have been detected. Additionally, the lack of linear retention indices and individual certified standards prevents the unequivocal identification of the detected compounds. However, the use of GC-MS, along with spectral fingerprint comparisons with widely validated libraries, is an accepted strategy for the tentative identification of metabolites in exploratory studies of complex plant matrices [43]. Additionally, some of the compounds reported by GC-MS may be common contaminants from GC columns, solvents, or plastic derivatives, possibly originating from the soil in which *F. trinervia* grows, such as some cyclosiloxane derivatives [41], for example, hexamethylcyclotrisiloxane. This raises the question of whether these are analytical artifacts or actual plant metabolites. In this context, the results obtained should be interpreted as an initial approach to prioritize compounds of biological interest, rather than as a comprehensive analytical characterization.

Furthermore, because the interaction predictions in this study were derived from computational analysis and because GC-MS primarily identifies volatile compounds, the disease-target associations may be biased toward widely studied pathologies. However, this is a starting point for exploring multiple treatment options for various diseases using *F. trinervia*.

However, despite the results obtained by combining GC-MS and network pharmacology analyses in this work, the molecular mechanisms underlying the pharmacodynamic effects of *Flaveria trinervia* remain to be fully characterized, and the findings should be interpreted within the limitations of an in silico, hypothesis-generating framework. Future studies should therefore prioritize: (i) incorporate certified analytical standards, retention indices, and complementary methodologies to definitively confirm the identity of the identified metabolites [44,45], (ii) the isolation and structural confirmation of key metabolites such as phytol, germacrene D, caryophyllene oxide, pinene isomers, squalene, and 2,2′:5′,2″-terthiophene; (iii) in vitro and in vivo validation in tumor- and immune-related models, with particular emphasis on the PI3K/AKT, JAK/STAT, and TNF signaling pathways highlighted in this study, and also ESR1, RXRA/B/G, NCOA2, and CYP19A1; and (iv) pharmacokinetic and toxicity assessments to define therapeutic windows, especially considering the reported dose-dependent cytotoxicity of phytol and related metabolites [24,43].

In addition, integrating multi-omics approaches, including transcriptomics, proteomics, and metabolomics, would enable system-level characterization of cellular responses and facilitate validation of predicted pathways across multiple regulatory layers [46,47]. Complementarily, chemical probe-based strategies offer powerful means to directly interrogate compound–target interactions. Target identification is essential for a comprehensive understanding of the therapeutic efficacy and molecular mechanisms of nanoparticles, for assessing off-target adverse effects, and for facilitating the optimization of drug structure and future drug design [48]. In particular, Proteolysis-Targeting Chimera (PROTAC) probe technologies provide highly selective tools for enabling functional validation of predicted targets. PROTAC strategies, such as TF-PROTAC, RNA-PROTAC, and BioPROTAC, have been developed using polypeptides, fusion proteins, and oligonucleotides as ligands to degrade undruggable targets, including MYC, KRAS, transcription factors (TFs), and RNA-binding proteins (RBPs); they exploit protein/protein or protein/nucleic acid interactions, allowing selective recruitment of E3 ligases and subsequent proteasomal degradation of disease-relevant targets [49]. Furthermore, the development of PROTAC strategies for Targeted Protein Degradation (TPD) has broadened the range of therapeutic targets, offering potential to overcome cancer resistance. Anti-proliferative proteins (APCs) combine aptamers and PROTACs, enhancing the tumor-specific targeting of traditional PROTACs [50]. On the other hand, integrating the cellular thermal shift assay (CETSA) with complementary approaches can substantially enhance the precision and efficiency of target identification for natural products, ultimately accelerating the development of therapeutic applications. CETSA exploits ligand-induced protein stabilization, in which ligand binding increases a protein’s thermal stability by reducing its conformational flexibility, enabling direct assessment of drug–target engagement without chemical modification of the ligand. Furthermore, integrating CETSA with advanced mass spectrometry techniques and high-throughput platforms has markedly expanded proteome coverage and analytical sensitivity, enabling the simultaneous quantification of thousands of proteins and facilitating the identification of low-abundance targets in native cellular environments [51].

The incorporation of these advanced methodologies will allow for refining the identification of molecular targets, strengthening the translational relevance of the findings, and ultimately guiding the experimental validation of the bioactive metabolites found in the leaves of *F. trinervia*.

In general, our integrative analysis supports the notion that *F. trinervia* is a promising source of multi-target phytochemicals with potential relevance in the treatment of tumors and immune-related disorders. The consistency between our network-based predictions and previously reported biological activities for the plant and its individual metabolites reinforces the pharmacological potential of *F. trinervia*. It provides a rational framework for subsequent experimental validation.

## 5. Conclusions

The comparative analysis of *Flaveria trinervia* leaf extracts revealed a diverse panel of metabolites with demonstrated or strongly suggested biological activity. Several of these compounds, particularly phytol, germacrene D, caryophyllene oxide, pinene isomers, squalene, and 2,2′:5′,2″-terthiophene, were associated with molecular targets involved in pathways relevant to cancer biology and immune regulation. The identification of central nodes such as ESR1, RXR isoforms, NCOA2, CYP19A1, EGFR, and MAPK1 highlights the importance of hormone-dependent and signal transduction pathways as key axes of action. The consistency of these observations with previously reported antioxidant, anti-inflammatory, and cytotoxic effects strengthens the pharmacological relevance of *F. trinervia* and supports its prioritization for further validation, rather than constituting direct evidence of therapeutic efficacy. In conclusion, we suggest that *F. trinervia* is a promising source of multi-target phytochemicals with broad system-level biological potential. Experimental studies focusing on these candidate metabolites, including mechanistic validation and pharmacokinetic evaluation, will be necessary to assess their therapeutic potential and explore their application across metabolic, immune, neurological, and cancer-related research areas. It is important to emphasize that the chemical identifications reported in this study are presumptive and serve as a foundation for subsequent analytical and biological validation. The incorporation of certified standards, retention indices, and complementary experimental approaches will enable definitive compound identification and a more robust assessment of their pharmacological relevance.

## Figures and Tables

**Table 1:** Main components of *F. trinervia* (Spreng.) C. Mohr leaf extract in a hexane/dichloromethane mixture was identified by GC-MS analysis.

No.	RT (min)	Area (%)	Compound in NIST	CAS Number	Similarity (%)
3	12.374	0.14	Cyclofenchene	488-97-1	91
5	13.845	0.05	beta-Thujene	28634-89-1	86
12	27.308	0.43	(-)-Isocaryophyllene	118-65-0	90
10	28.870	0.07	Germacrene D	23986-74-5	91
18	31.485	0.29	Caryophyllene oxide	1139-30-6	90
38	44.657	1.59	2,2′:5′,2″-Terthiophene	1081-34-1	98

Abbreviations: RT, retention time; CAS, Chemical Abstracts Service; NIST, National Institute of Standards and Technology.

**Table 2:** Main components of *F. trinervia* (Spreng.) C. Mohr leaf extract in an acetone/chloroform mixture was identified by GC-MS analysis.

No.	RT (min)	Area (%)	Compound in NIST	CAS Number	Similarity (%)
4	12.385	0.82	(+/-)-alpha-Pinene	80-56-8	94
5	13.856	0.30	beta-Pinene	18172-67-3	91
6	27.308	1.55	(-)-Caryophyllene	87-44-5	95
33	42.094	0.74	Phytol	150-86-7	91
36	44.651	5.57	2,2′:5′,2″-Terthiophene	1081-34-1	97
55	53.698	0.97	Squalene	111-02-4	86

Abbreviations: RT, retention time; CAS, Chemical Abstracts Service; NIST, National Institute of Standards and Technology.

**Table 3:** Key targets of extract components from *Flaveria trinervia* (Spreng.) C. Mohr leaves and their relevant topological parameters.

Uniprot ID	Protein Name	Gene Name	Degree	* ASPL	* CC	* BC
P12931	Proto-oncogene tyrosine-protein kinase Src	SRC	16	3.483050847457627	0.2871046228710462	0.462478281230358
Q15596	Nuclear receptor coactivator 2	NCOA2	15	3.720338983050847	0.26879271070615035	0.37310083944795197
P19793	Retinoic acid receptor RXR-alpha	RXRA	15	4.271186440677966	0.23412698412698413	0.08992741348878065
P03372	Estrogen receptor	ESR1	13	3.355932203389830	0.297979797979798	0.2150328605201165
P28702	Retinoic acid receptor RXR-beta	RXRB	11	4.449152542372881	0.22476190476190475	0.02473434345638247
P28482	Mitogen-activated protein kinase 1	MAPK1	10	3.864406779661017	0.2776470588235294	0.24469094190315152
						
P08238	Heat shock protein HSP 90-beta	HSP90AB1	10	3.86440677966101	0.25877192982456143	0.057876390254256664
P48443	Retinoic acid receptor RXR-gamma	RXRG	9	4.491525423728813	0.2226415094339623	0.010437563589645798
P11511	Aromatase	CYP19A1	8	4.1440677966101696	0.2413087934560327	0.12774755062890666
Q16665	Hypoxia-inducible factor 1-alpha	HIF1A	8	4.0508474576271185	0.24686192468619247	0.05503076647144445
P00533	Epidermal growth factor receptor	EGFR	8	3.906779661016949	0.2559652928416486	0.015836057643967248

Abbreviations: Average Shortest Path Length, ASPL; Closeness Centrality, CC; Betweenness Centrality, BC. * ASPL: a low ASPL indicates that proteins (nodes) are more interconnected, suggesting a more compact network with greater efficiency in information transmission, while a high ASPL reflects that, on average, proteins are more distant from each other, which may indicate a more fragmented or less integrated network. * CC: A node with high closeness centrality can reach all other nodes more quickly, acting as an efficient point for communication and signal propagation. In contrast, a node with low closeness centrality is more peripheral and requires more steps to connect with the rest of the network. * BC: a node with high betweenness centrality functions as a critical connector or “bottleneck,” controlling the flow of information between different regions of the network, while nodes with low betweenness centrality play a minor role in global communication, as they rarely lie on shortest paths between other nodes.

**Table 4:** Tumor-related diseases obtained from DISGENET based on the top 11 PPI network targets.

Compound Identifier	Compound Name	Associated Disease	Target
No. 1 No. 2	Cyclofenchene beta-Thujene	Breast Neoplasms	ESR1 CYP19A1
		Carcinoma	ESR1
		Prostatic Neoplasms	
		Endometrial Neoplasms	CYP19A1
No. 3 No. 8 No. 9	(-)-Isocaryophyllene beta.-Pinene Caryophyllene	Breast Neoplasms	NCOA2 ESR1 CYP19A1
		Prostatic Neoplasms	NCOA2 ESR1
		Acute Myeloid Leukemia	NCOA2
		Colorectal Neoplasms	
		Non-Small Cell Lung Carcinoma	
		Mesenchymal Chondrosarcoma	
		Rhabdomyosarcoma	
		Carcinoma	ESR1
		Endometrial Neoplasms	CYP19A1
No. 4	Germacrene D	Breast Neoplasms	NCOA2 ESR1 RXRB CYP19A1
		Prostatic Neoplasms	NCOA2 RXRA ESR1
		Non-Small Cell Lung Carcinoma	NCOA2 RXRG
		Thyroid Neoplasms	RXRA MAPK1
		Acute Myeloid Leukemia	NCOA2
		Colorectal Neoplasms	
		Mesenchymal Chondrosarcoma	
		Rhabdomyosarcoma	
		Pancreatic Neoplasms	RXRA
		Multiple Myeloma	
		Carcinoma	ESR1
		Stomach Neoplasms	RXRB
		Melanoma	MAPK1
		Renal Cell Carcinoma	
		Ovarian Neoplasms	
		Squamous Cell Carcinoma	
		Lung Neoplasms	
		Papillary Thyroid Carcinoma	RXRG
		Endometrial Neoplasms	CYP19A1
No. 5	Caryophyllene oxide	Breast Neoplasms	NCOA2 CYP19A1 HIF1A
		Prostatic Neoplasms	NCOA2 HIF1A
		Non-Small Cell Lung Carcinoma	
		Acute Myeloid Leukemia	NCOA2
		Colorectal Neoplasms	
		Mesenchymal Chondrosarcoma	
		Rhabdomyosarcoma	
		Endometrial Neoplasms	CYP19A1
		Glioblastoma	HIF1A
		Carcinoma	
No. 6 No. 11	2,2′:5′,2″-Terthiophene Squalene	Breast Neoplasms	SRC CYP19A1
		Colorectal Neoplasms	SCR
		Colonic Neoplasms	
		Urinary Bladder Neoplasms	
		Liver Neoplasms	HSP90AB1
		Benign Neoplasm	
		Endometrial Neoplasms	CYP19A1
		Non-Small Cell Lung Carcinoma	EGFR
		Lung Adenocarcinoma	
		Glioblastoma	
		Squamous Cell Carcinoma	
		Ovarian Neoplasms	
		Lung Neoplasms	
		Adenocarcinoma	
		Carcinoma	
No. 7	(+/-)-alpha-Pinene	Prostatic Neoplasms	RXRA ESR1
		Breast Neoplasms	ESR1 RXRB CYP19A1
		Thyroid Neoplasms	RXRA MAPK1
		Pancreatic Neoplasms	RXRA
		Multiple Myeloma	
		Stomach Neoplasms	RXRB
		Melanoma	MAPK1
		Renal Cell Carcinoma	
		Ovarian Neoplasms	
		Squamous Cell Carcinoma	
		Lung Neoplasms	
		Non-Small Cell Lung Carcinoma	RXRG
		Papillary Thyroid Carcinoma	
		Hypogonadism	CYP19A1
No. 10	Phytol	Prostatic Neoplasms	RXRA ESR1
		Carcinoma	ESR1 EGFR
		Thyroid Neoplasms	RXRA
		Multiple Myeloma	
		Breast Neoplasms	ESR1
		Non-Small Cell Lung Carcinoma	EGFR
		Lung Adenocarcinoma	
		Glioblastoma	
		Squamous Cell Carcinoma	
		Ovarian Neoplasms	
		Lung Neoplasms	
		Adenocarcinoma	

Abbreviations: ESR1, Estrogen Receptor 1; CYP19A1, Cytochrome P450 Family 19 Subfamily A Member 1 (aromatase); NCOA2, Nuclear Receptor Coactivator 2 (SRC-2/GRIP1); RXRA, Retinoid X Receptor Alpha; RXRB, Retinoid X Receptor Beta; RXRG, Retinoid X Receptor Gamma; MAPK1, Mitogen-Activated Protein Kinase 1 (ERK2); HIF1A, Hypoxia-Inducible Factor 1 Alpha; SRC, Proto-oncogene Tyrosine-Protein Kinase Src; HSP90AB1, Heat Shock Protein 90 Alpha Family Class B Member 1; EGFR, Epidermal Growth Factor Receptor.

**Table 5:** Immune-related diseases obtained from DISGENET based on the top 11 PPI network targets.

Compound Identifier	Compound Name	Associated Disease	Target
No. 4	Germacrene D	Systemic Lupus Erythematosus	RXRB
		Granulomatosis with Polyangiitis	
No. 6 No. 11	2,2′:5′,2″-Terthiophene Squalene	Multiple Sclerosis	HSP90AB1
		HIV Infections	
		Asthma	
No. 7	(+/-)-alpha-Pinene	Systemic Lupus Erythematosus	RXRB

Abbreviations: HSP90AB1, Heat Shock Protein 90 Alpha Family Class B Member 1; RXRB, Retinoid X Receptor Beta.

**Table 6:** Metabolic & Endocrine-related diseases obtained from DISGENET based on the top 11 PPI network targets.

Compound Identifier	Compound Name	Associated Disease	Target
No. 1 No. 2	Cyclofenchene beta-Thujene	Osteoporosis	ESR1 CYP19A1
		Endometriosis	
		Estrogen Resistance	ESR1
		Obesity	
		Aromatase Excess Syndrome	CYP19A1
		Polycystic Ovary Syndrome	
		Aromatase Deficiency	
		Hypogonadism	
No. 3 No. 8 No. 9	(-)-Isocaryophyllene beta.-Pinene Caryophyllene	Osteoporosis	ESR1 CYP19A1
		Endometriosis	
		Estrogen Resistance	ESR1
		Obesity	
		Aromatase Excess Syndrome	
		Polycystic Ovary Syndrome	CYP19A1
		Aromatase Deficiency	
		Hypogonadism	
No. 4	Germacrene D	Obesity	RXRA ESR1 RXRG
		Osteoporosis	ESR1 CYP19A1
		Endometriosis	
		Estrogen Resistance	ESR1
		Metabolic Syndrome	RXRG
		Dyslipidemias	
		Thyroid Hormone Resistance Syndrome	
		Aromatase Excess Syndrome	CYP19A1
		Polycystic Ovary Syndrome	
		Aromatase Deficiency	
		Hypogonadism	
No. 5	Caryophyllene oxide	Endometriosis	CYP19A1
		Osteoporosis	
		Aromatase Excess Syndrome	
		Polycystic Ovary Syndrome	
		Aromatase Deficiency	
		Hypogonadism	
		Diabetes Mellitus, Type 2	HIF1A
No. 6 No. 11	2,2′:5′,2″-Terthiophene Squalene	Osteoporosis	SRC CYP19A1
		Endometriosis	CYP19A1
		Aromatase Excess Syndrome	
		Polycystic Ovary Syndrome	
		Aromatase Deficiency	
		Hypogonadism	
No. 7	(+/-)-alpha-Pinene	Obesity	RXRA ESR1 RXRG
		Osteoporosis	ESR1 CYP19A1
		Estrogen Resistance	ESR1
		Metabolic Syndrome	RXRG
		Dyslipidemias	
		Thyroid Hormone Resistance Syndrome	
		Aromatase Excess Syndrome	CYP19A1
		Polycystic Ovary Syndrome	
		Aromatase Deficiency	
		Hypogonadism	
No. 10	Phytol	Obesity	RXRA ESR1
		Osteoporosis	ESR1
		Endometriosis	
		Estrogen Resistance	

Abbreviations: ESR1, Estrogen Receptor 1; CYP19A1, Cytochrome P450 Family 19 Subfamily A Member 1 (aromatase); RXRA, Retinoid X Receptor Alpha; RXRG, Retinoid X Receptor Gamma; HIF1A, Hypoxia-Inducible Factor 1 Alpha; SRC, Proto-oncogene Tyrosine-Protein Kinase Src.

**Table 7:** Neurological and psychiatric-related diseases obtained from DISGENET based on the top 11 PPI network targets.

Compound Identifier	Compound Name	Associated Disease	Target
No. 1 No. 2	Cyclofenchene beta-Thujene	Alzheimer Disease	ESR1 CYP19A1
No. 3 No. 8 No. 9	(-)-Isocaryophyllene beta.-Pinene Caryophyllene	Alzheimer Disease	ESR1 CYP19A1
No. 4	Germacrene D	Alzheimer Disease	ESR1 CYP19A1
		Schizophrenia	RXRB MAPK1
		Cognitive Dysfunction	RXRB
		Stroke	
		Memory Disorders	
		Parkinson Disease	MAPK1
		Intellectual Disability	
No. 5	Caryophyllene oxide	Alzheimer Disease	CYP19A1
No. 6 No. 11	2,2′:5′,2″-Terthiophene Squalene	Neurodegenerative Diseases	HSP90AB1
		Alzheimer Disease	CYP19A1
No. 7	(+/-)-alpha-Pinene	Alzheimer Disease	ESR1 CYP19A1
		Schizophrenia	RXRB MAPK1
		Cognitive Dysfunction	RXRB
		Stroke	
		Memory Disorders	
		Parkinson Disease	MAPK1
		Intellectual Disability	
No. 10	Phytol	Alzheimer Disease	ESR1

Abbreviations: ESR1, Estrogen Receptor 1; CYP19A1, Cytochrome P450 Family 19 Subfamily A Member 1 (aromatase); RXRB, Retinoid X Receptor Beta; MAPK1, Mitogen-Activated Protein Kinase 1 (ERK2); HSP90AB1, Heat Shock Protein 90 Alpha Family Class B Member 1.

**Table 8:** Cardiovascular-related diseases obtained from DISGENET based on the top 11 PPI network targets.

Compound Identifier	Compound Name	Associated Disease	Target
No. 4	Germacrene D	Heart Defects, Congenital	RXRA
		Atherosclerosis	RXRB
		Myocardial Ischemia	RXRG
No. 5	Caryophyllene oxide	Myocardial Infarction	HIF1A
		Hypertension	
No. 6 No. 11	2,2′:5′,2″-Terthiophene Squalene	Hypertension	HSP90AB1 EGFR
No. 7	(+/-)-alpha-Pinene	Heart Defects, Congenital	RXRA
		Atherosclerosis	RXRB
		Myocardial Ischemia	RXRG
No. 10	Phytol	Heart Defects, Congenital	RXRA
		Hypertension	EGFR

Abbreviations: EGFR, Epidermal Growth Factor Receptor; HIF1A, Hypoxia-Inducible Factor 1 Alpha; HSP90AB1, Heat Shock Protein 90 Alpha Family Class B Member 1; RXRA, Retinoid X Receptor Alpha; RXRB, Retinoid X Receptor Beta; RXRG, Retinoid X Receptor Gamma.

## Data Availability

The raw data supporting the conclusions of this article will be made available by the authors on request.

## Supplementary Materials

The following supporting information can be downloaded at: https://www.mdpi.com/article/10.3390/cimb48020160/s1.

## Abbreviations

ASPL	Average Shortest Path Length
BC	Betweenness Centrality
CAS	Chemical Abstracts Service
CC	Closeness Centrality
FDR	False Discovery Rate
*F. trinervia*	*Flaveria trinervia*
GC-MS	Gas chromatography–mass spectrometry
GO	Gene Ontology
IMPPAT	Indian Medicinal Plants, Phytochemistry and Therapeutics
KEGG	Kyoto Encyclopedia of Genes and Genomes
PPI	Protein–protein interaction
RT	Retention time
