# Supplementary material for: From Gas Chromatography–Mass Spectrometry (GC–MS) to Network Pharmacology: System-Level Insights into the Multi-Target Biological Potential of Flaveria trinervia (Spreng.) C. Mohr
Source: Curr Issues Mol Biol. 2026 Feb 1;48(2):160. doi: 10.3390/cimb48020160 (PMC12939618; doi:10.3390/cimb48020160)
Supplement: Supplementary file 1 [file cimb-48-00160-s001.zip › Material suplementario/Spectrum.pdf]

POLVO 2

toxi   Library Search Report

Data Path : D:\vsk\MARZO\

Data File : 230314-07.D

Acq On   : 10 Mar 2023 15:01 (#1); 10 Mar 2023 14:54 (#2)

Operator : KELVIN/KAREN/ALE

Sample   : POLVO 2

Misc   : POLVO 2 (Sig #1); (Sig #2)

ALS Vial : 5   Sample Multiplier: 1

Search Libraries: C:\Database\NIST98.L   Minimum Quality: 0

C:\Database\WILEY275.L   Minimum Quality: 0

Unknown Spectrum: Apex

Integration Events: ChemStation Integrator - autoint1.e

Pk#   RT Area%   Library/ID   Ref#   CAS#   Qual

---

1   7.825 0.18 C:\Database\NIST98.L

Toluene   117292 000108-88-3 90

Toluene   37326 000108-88-3 83

Toluene   117282 000108-88-3 83

2   8.409 0.36 C:\Database\NIST98.L

3-Penten-2-one, 4-methyl- 116425 000141-79-7 86

Furan, 2,5-dihydro-2,5-dimethyl- 32804 059242-27-2 80

3-Penten-2-one, 4-methyl- 116450 000141-79-7 80

3 9.553 1.37 C:\Database\NIST98.L

Butane, 1-ethoxy- 113142 000628-81-9 9

Butane, 1-ethoxy- 113143 000628-81-9 9

Propane, 1-ethoxy-2-methyl- 19286 000627-02-1 9

4 12.385 0.82 C:\Database\NIST98.L

1R-.alpha.-Pinene 40147 007785-70-8 94

Bicyclo[3.1.1]hept-2-ene, 2,6,6-tr 40144 002437-95-8 91

imethyl-, (.+/-.)-

Tricyclo[2.2.1.02,6]heptane, 1,3,3 117890 000488-97-1 91

-trimethyl-

5 13.856 0.30 C:\Database\NIST98.L

.beta.-Pinene 117800 000127-91-3 91

Bicyclo[3.1.0]hex-2-ene, 4-methyl- 40029 028634-89-1 91

1-(1-methylethyl)-

Cyclohexene, 4-methylene-1-(1-meth 40025 000099-84-3 91

ylethyl)-

6 27.308 1.55 C:\Database\NIST98.L

Caryophyllene 108831 000087-44-5 95

Bicyclo[5.2.0]nonane, 2-methylene- 40354 1000159-38-9 93

4,8,8-trimethyl-4-vinyl-

Bicyclo[7.2.0]undec-4-ene, 4,11,11 108830 000118-65-0 91

-trimethyl-8-methylene-,[1R-(1R*,4

Z,9S*)]-

7 28.870 0.24 C:\Database\NIST98.L

Germacrene D 124226 023986-74-5 58

5,6-Decadien-3-yne, 5,7-diethyl- 71008 061227-89-2 47

1H-Cyclopropa[a]naphthalene, 1a,2, 70998 017334-55-3 43

3,5,6,7,7a,7b-octahydro-1,1,7,7a-t

etramethyl-, [1aR-(1a.alpha.,7.alp

ha.,7a.alpha.,7b.alpha.)]-

8 29.196 0.26 C:\Database\NIST98.L

2,4,5,5,8a-Pentamethyl-6,7,8,8a-te 80695 1000195-40-9 83

trahydro-5H-chromene

Phenol, 2,5-bis(1,1-dimethylethyl) 125777 005875-45-6 72

Phenol, 2,4-bis(1,1-dimethylethyl) 125782 000096-76-4 64

9 30.169 0.35 C:\Database\NIST98.L

2(4H)-Benzofuranone, 5,6,7,7a-tetr 120085 017092-92-1 83

ahydro-4,4,7a-trimethyl-, (R)-

2(4H)-Benzofuranone, 5,6,7,7a-tetr 50182 015356-74-8 81

ahydro-4,4,7a-trimethyl-

2(4H)-Benzofuranone, 5,6,7,7a-tetr 120127 015356-74-8 53
ahydro-4,4,7a-trimethyl-

10 31.314 0.27 C:\Database\NIST98.L
Methanesulfenyl chloride, trichlor 67025 000594-42-3 16
o-
Tolazoline acetate 38629 1000119-76-1 11
Benzaldehyde, 4-methyl-, O-methylo 38449 033499-39-7 9
xime

11 31.485 1.02 C:\Database\NIST98.L
Caryophyllene oxide 109343 001139-30-6 68
2,6-Dimethyl-1,3,6-heptatriene 37131 000928-67-6 50
7-Propylidene-bicyclo[4.1.0]heptan 30826 1000190-24-0 43

12 32.761 0.38 C:\Database\NIST98.L
2,5-Methano-1H-indene, octahydro- 122495 019026-94-9 47
Adamantane 122490 000281-23-2 46
5-Methylene-1,3a,4,5,6,6a-hexahydr 61840 1000193-00-3 45
opentalen-1-ol

13 33.167 0.29 C:\Database\NIST98.L
8-Heptadecene 13511 054290-12-9 86
1-Hexyl-1-nitrocyclohexane 14299 1000143-77-5 47
3-Heptadecene, (Z)- 13158 1000141-67-3 47

14 33.471 0.45 C:\Database\NIST98.L

3-Buten-2-one, 4-(3-hydroxy-6,6-di   4600 1000142-31-3 50

methyl-2-methylenecyclohexyl)-

3-buten-2-one, 4-(5,5-dimethyl-1-o   6062 1000196-66-5 38

xaspiro[2.5]oct-4-yl)

Ingol 12-acetate   4568 1000203-01-6 27

15 33.820 0.45 C:\Database\NIST98.L

Diethyl chloromethanephosphonate   119887 003167-63-3 35

Diethyl chloromethanephosphonate   49351 003167-63-3 35

trans-Z-.alpha.-Bisabolene epoxide   4198 1000131-71-1 22

16 34.249 0.37 C:\Database\NIST98.L

1,1,1,5,7,7,7-Heptamethyl-3,3-bis(26950 038147-00-1 38

trimethylsiloxy)tetrasiloxane

Cyclononasiloxane, octadecamethyl- 115158 000556-71-8 25

1h-Pyrrole-3,4-diacetic acid, 2-ac 97361 1000195-85-5 10

etoxymethyl-5-methoxycarbonyl-, di

methyl ester

17 34.478 1.74 C:\Database\NIST98.L

Cyclononasiloxane, octadecamethyl- 115158 000556-71-8 72

3-Isopropoxy-1,1,1,7,7,7-hexamethy 26784 071579-69-6 35

l-3,5,5-tris(trimethylsiloxy)tetra

siloxane

3,6-Dioxa-2,4,5,7-tetrasilaoctane, 87890 004342-25-0 16

2,2,4,4,5,5,7,7-octamethyl-

18 34.524 0.49 C:\Database\NIST98.L

N-Deacetyl-N-ethoxycarbonylcolchic 106132 1000127-08-4 25

ine

Cholestane, 3-thiocyanato-, (3.alp 5251 020997-49-3 10

ha.,5.alpha.)-

Flavone, 4',5,7-triethoxy-3,3',6-t 106124 014397-67-2 10

rimethoxy-

19 34.569 1.26 C:\Database\NIST98.L

Cyclodecasiloxane, eicosamethyl- 27712 018772-36-6 46

1,1,1,5,7,7,7-Heptamethyl-3,3-bis(26950 038147-00-1 40

trimethylsiloxy)tetrasiloxane

Cyclononasiloxane, octadecamethyl- 115158 000556-71-8 33

20 34.690 0.95 C:\Database\NIST98.L

Cyclononasiloxane, octadecamethyl- 115158 000556-71-8 68

Silane, [[4-[1,2-bis[(trimethylsil 128841 056114-62-6 35

yl)oxy]ethyl]-1,2-phenylene]bis(ox

y)]bis[trimethyl-

Silane, [[4-[1,2-bis[(trimethylsil 128842 056114-62-6 35

yl)oxy]ethyl]-1,2-phenylene]bis(ox

y)]bis[trimethyl-

21 34.781 0.21 C:\Database\NIST98.L

Cyclodecasiloxane, eicosamethyl- 27712 018772-36-6 43

1,1,1,5,7,7,7-Heptamethyl-3,3-bis(26950 038147-00-1 42

trimethylsiloxy)tetrasiloxane

Trisiloxane, 1,1,1,5,5,5-hexamethy 115144 003555-47-3 27

l-3,3-bis[(trimethylsilyl)oxy]-

22 35.216 4.75 C:\Database\NIST98.L

Cyclononasiloxane, octadecamethyl- 115158 000556-71-8 38

Pentasiloxane, dodecamethyl- 115143 000141-63-9 22

Trisiloxane, 1,1,1,5,5,5-hexamethy 26961 003555-47-3 22

l-3,3-bis[(trimethylsilyl)oxy]-

23 35.382 4.85 C:\Database\NIST98.L

Cyclononasiloxane, octadecamethyl- 115158 000556-71-8 42

2H-1,4-Benzodiazepin-2-one, 7-chlo 28052 055319-93-2 25

ro-1,3-dihydro-5-phenyl-1-(trimeth

ylsilyl)-3-[(trimethylsilyl)oxy]-

3,6-Dioxa-2,4,5,7-tetrasilaoctane, 87890 004342-25-0 16

2,2,4,4,5,5,7,7-octamethyl-

24 35.679 0.76 C:\Database\NIST98.L

Cyclononasiloxane, octadecamethyl- 115158 000556-71-8 14

Cyclohexyl-(hexahydro-benzofuran-2 87902 1000190-83-1 10

-ylidene)-amine

3,5,7-Trimethyl-1-azaadamantan-4,6 87851 1000216-21-5 9

,10-trione

25 36.635 1.66 C:\Database\NIST98.L

1,19-Eicosadiene   13201 014811-95-1 45

Bicyclo[3.1.1]heptane, 2,6,6-trime   2145 004863-59-6 41

thyl-, [1R-(1.alpha.,2.alpha.,5.al

pha.)]-

Bicyclo[3.1.1]heptane, 2,6,6-trime   2052 004795-86-2 41

thyl-, [1R-(1.alpha.,2.beta.,5.alp

ha.)]-

26 36.767 0.59 C:\Database\NIST98.L

3-Methyl-3-(N-methyl-2-pyrrolyl)-1 97901 086738-93-4 22

,2-diphenylcyclopropene

2,4,6(1H,3H,5H)-Pyrimidinetrione,   97842 057346-58-4 16

5-ethyl-1,3-dimethyl-5-[2-[(trimet

hylsilyl)oxy]ethyl]-

Benzene, 1-methyl-2-(2-propenyl)-   52460 001587-04-8 14

27 37.144 0.41 C:\Database\NIST98.L

Bicyclo[2.2.1]heptane, 1,3,3-trime 32120 006248-88-0 49

thyl-

Cyclohexanol, 5-methyl-2-(1-methyl 2373 000089-79-2 38
ethenyl)-, [1R-(1.alpha.,2.beta.,5
.alpha.)]-
1,2-Dioctylcyclopropene 31479 001089-40-3 38

28 37.539 0.59 C:\Database\NIST98.L
1,2-Dihexylcyclopropene 31740 035365-52-7 46
N-(1-Cyano-3-methyl-but-2-enyl)-ac 6907 1000192-62-7 38
etamide
Oleyl Alcohol 14152 000143-28-2 30

29 38.277 0.97 C:\Database\NIST98.L
.alpha.-Cedrene oxide 84384 1000159-39-1 55
Ethanone, 1-(5,6,7,8-tetrahydro-2, 84211 071596-88-8 49
8,8-trimethyl-4H-cyclohepta[b]fura
n-5-yl)-
1,3-Pentadiene, 1,1-diphenyl-, (Z) 126412 015295-31-5 41

30 38.832 0.26 C:\Database\NIST98.L
Thianthrene 126750 000092-85-3 90
Thianthrene 86759 000092-85-3 87
Thianthrene 126748 000092-85-3 64

31 39.181 0.50 C:\Database\NIST98.L
n-Hexadecanoic acid 109984 000057-10-3 42

n-Hexadecanoic acid 109985 000057-10-3 42

n-Hexadecanoic acid 114901 000057-10-3 38

32 40.726 1.24 C:\Database\NIST98.L

Hexadecanoic acid, tert-butyldimet 100671 1000221-81-6 37

hylsilyl ester

Hexadecanoic acid, trimethylsilyl 128570 055520-89-3 22

ester

Benzene, (2-methyl-2-propenyl)- 52561 003290-53-7 14

33 42.094 0.74 C:\Database\NIST98.L

Phytol 114590 000150-86-7 91

Phytol 114545 000150-86-7 80

Phytol 114589 000150-86-7 72

34 42.603 1.30 C:\Database\NIST98.L

9-Octadecenoic acid, (E)- 111528 000112-79-8 93

9-Octadecenoic acid, (E)- 13227 000112-79-8 86

Oleic Acid 1968 000112-80-1 60

35 43.902 0.50 C:\Database\NIST98.L

Oleic acid, trimethylsilyl ester I 28765 1000141-36-2 33

Oleic acid, trimethylsilyl ester 120456 021556-26-3 32

(1H)Indolo[2,1-a]isoquinoline, 11, 102585 1000124-45-2 27

12-dihydro-2,3,8,9-tetramethoxy-

36 44.651 5.57 C:\Database\NIST98.L

2,2':5',2''-Terthiophene 92802 001081-34-1 97

2,2':4',2''-Terthiophene 92714 021732-47-8 94

2,2':5',2''-Terthiophene 127600 001081-34-1 94

37 46.397 0.26 C:\Database\NIST98.L

Bicyclo[3.3.1]nonan-9-ol, 1-phenyl 86723 036399-43-6 38

Hydrazidiamantane 86707 1000138-39-6 38

Pyrene, 1,2,3,3A,4,5,5A,6,7,8,8A,9 86763 126188-35-0 35

,10,10A-tetradecahydro-

38 47.879 26.74 C:\Database\NIST98.L

Cyclononasiloxane, octadecamethyl- 115158 000556-71-8 72

Cyclodecasiloxane, eicosamethyl- 27712 018772-36-6 41

3,6-Dioxa-2,4,5,7-tetrasilaoctane, 87890 004342-25-0 30

2,2,4,4,5,5,7,7-octamethyl-

39 48.296 0.22 C:\Database\NIST98.L

Tricyclo[10.2.2.25,8]octadeca-5,7, 52694 024777-32-0 25

12,14,15,17-hexaene, 6-nitro-

Tricyclo[6.6.0.0(3,6)]tetradeca-1(52396 1000151-40-9 16

8),4,11-triene

Thiophene, tetrahydro-3-phenyl-, 1 59271 093134-22-6 14

-oxide

40 48.365 0.33 C:\Database\NIST98.L

9-Octadecenoic acid (Z)-, 2-hydrox 13221 003443-84-3 18

y-1-(hydroxymethyl)ethyl ester

5-Acetoxypentadecane 4235 1000245-62-3 14

9-Octadecenoic acid, (E)- 13227 000112-79-8 10

41 48.685 0.22 C:\Database\NIST98.L

9-Octadecenoic acid (Z)-, 2-hydrox 13017 004500-01-0 42

yethyl ester

9-Octadecenoic acid (Z)-, 2,3-dihy 13237 000111-03-5 38

droxypropyl ester

9-Octadecenoic acid, (E)- 111528 000112-79-8 35

42 49.881 0.16 C:\Database\NIST98.L

.beta.-D-Glucopyranose, 2-O-(3-O-a 8650 063166-73-4 14

cetyl-6-deoxy-.beta.-D-glucopyrano

syl)-6-deoxy-, 3-acetate 1-(decahy

dro-5''-methyl-4''-((1-oxo-3-pheny

l-2- propenyl)oxy)dispiro(oxirane-

2,3'(2'H)-benzofuran-2',2''- (2H)p

yran)-6'-carboxylate) (2'S-(2'.alp

ha.(4''R*(E),5''S

1,4-Di-O-acetyl-2,3,5-tri-O-methyl 52552 084925-40-6 14

ribitol

Tricyclo[10.2.2.25,8]octadeca-5,7, 52694 024777-32-0 11

12,14,15,17-hexaene, 6-nitro-

43 49.996 0.87 C:\Database\NIST98.L

Acetic acid, 3-methoxy-7-methyl-6, 90006 1000189-21-8 47

7,8,9-tetrahydro-dibenzofuran-2-yl

ester

Phenoxathiin, 10,10-dioxide 89951 000950-47-0 35

Azulene, 4,8-dimethyl-6-phenyl- 90029 042758-88-3 30

44 50.179 0.23 C:\Database\NIST98.L

Chromium, [(1,2,3,4,5,6-.eta.)-1,3 12524 043138-04-1 27

,5-cycloheptatriene](.eta.5-2,4-cy

clopentadien-1-yl)-

11-Hexadecenoic acid, 15-methyl-, 13883 055044-54-7 25

methyl ester

Oleic acid, trimethylsilyl ester 120456 021556-26-3 25

45 50.511 0.35 C:\Database\NIST98.L

2-Benzylsuccinic acid, diethyl est 52655 1000196-24-0 27

er

1,4-Di-O-acetyl-2,3,5-tri-O-methyl 52552 084925-40-6 25

ribitol

Oleic acid, trimethylsilyl ester 120456 021556-26-3 16

46 50.814 0.29 C:\Database\NIST98.L

Chromium, [(1,2,3,4,5,6-.eta.)-1,3 12524 043138-04-1 14

,5-cycloheptatriene](.eta.5-2,4-cy

clopentadien-1-yl)-

Galactitol, 1,3,5-tri-O-methyl-, t 8276 019318-48-0 14

riacetate

Tetrahydro-1,3-oxazine-2-thione 120443 017374-18-4 10

47 51.066 0.71 C:\Database\NIST98.L

1H-Indene, 2,3-dihydro-4-propyl- 59274 092013-16-6 43

2-Butanone, 4-[2-(1-methylethyl)-5 59088 1000197-43-1 15

-methyl-5-(2-oxo-5-methylcyclopent

ylmethyl)-1-cyclopentenyl]-

Benzene, (1-methyl-1-butenyl)- 59184 053172-84-2 14

48 51.129 0.40 C:\Database\NIST98.L

Thiophene, tetrahydro-3-phenyl-, 1 59271 093134-22-6 27

-oxide

Acetic acid, trifluoro-, 5,6,7,8,9 59345 064129-26-6 27

,10-hexahydro-7,7-dimethyl-10-oxo-

5-benzocyclooctenyl ester

1,4-Methanonaphthalen-9-ol, 1,2,3, 59290 001198-20-5 22

4-tetrahydro-, stereoisomer

49 51.220 0.26 C:\Database\NIST98.L

9-Octadecenoic acid, (E)- 111554 000112-79-8 25

Oleic acid, trimethylsilyl ester 120456 021556-26-3 25

9-Octadecenoic acid, (2-phenyl-1,3 109489 056599-45-2 25

-dioxolan-4-yl)methyl ester, cis-

50 51.432 0.70 C:\Database\NIST98.L

Tricyclo[10.2.2.25,8]octadeca-5,7, 52694 024777-32-0 25

12,14,15,17-hexaene, 6-nitro-

Hexanoic acid, 10-undecen-1-yl est 4208 1000160-13-4 16

er

1,4-Di-O-acetyl-2,3,5-tri-O-methyl 52552 084925-40-6 16

ribitol

51 51.764 1.21 C:\Database\NIST98.L

Benzene, 2-[(tert-butyldimethylsil 84728 1000221-75-3 27

yl)oxy]-1-isopropyl-4-methyl-

2-Methyl-5,5-diphenyl-4-(methylthi 84809 024133-96-8 18

o)imidazole

Indole-2-one, 2,3-dihydro-N-hydrox 84802 1000129-52-1 18

y-4-methoxy-3,3-dimethyl-

52 52.582 23.06 C:\Database\NIST98.L

Cyclononasiloxane, octadecamethyl- 115158 000556-71-8 62

Cyclodecasiloxane, eicosamethyl- 27712 018772-36-6 49

Silane, [[4-[1,2-bis[(trimethylsil 103450 056114-62-6 27

yl)oxy]ethyl]-1,2-phenylene]bis(ox
y)]bis[trimethyl-

53 52.817 0.38 C:\Database\NIST98.L
1,2-Bis(trimethylsilyl)benzene 84839 017151-09-6 35
Tricyclo[10.2.2.25,8]octadeca-5,7, 52694 024777-32-0 25
12,14,15,17-hexaene, 6-nitro-
4-Methoxy-6-methyl-5-nitroisobenzo 84705 089586-40-3 25
furan-1,3-dione

54 53.183 0.56 C:\Database\NIST98.L
9-Octadecenoic acid, (E)- 111554 000112-79-8 56
9-Octadecenoic acid, (E)- 13227 000112-79-8 46
9-Octadecenoic acid, (E)- 111528 000112-79-8 45

55 53.698 0.97 C:\Database\NIST98.L
2,6,10,14,18-Pentamethyl-2,6,10,14 23038 075581-03-2 95
,18-eicosapentaene
Squalene 114269 007683-64-9 86
3,7,11-Tridecatrienoic acid, 4,8,1 23071 036237-70-4 70
2-trimethyl-, methyl ester, (Z,E)-

56 54.139 0.65 C:\Database\NIST98.L
Tricyclo[10.2.2.25,8]octadeca-5,7, 52694 024777-32-0 49
12,14,15,17-hexaene, 6-nitro-

Benzene, 2-[(tert-butyldimethylsil 84728 1000221-75-3 30
yl)oxy]-1-isopropyl-4-methyl-
Silicic acid, diethyl bis(trimethy 84851 003555-45-1 27
lsilyl) ester

57 54.367 1.71 C:\Database\NIST98.L
Anthracene, 9,10-dihydro-9,9,10-tr 84844 014923-29-6 11
imethyl-
1H-Pyrrole-2,5-dione, 1-(4-chlorop 84714 001631-29-4 11
henyl)-
2-Myristynoyl-glycinamide 84715 1000111-57-7 11

58 54.785 0.63 C:\Database\NIST98.L
9-Octadecenoic acid, (E)- 13227 000112-79-8 35
9-Octadecenoic acid, (E)- 111554 000112-79-8 25
9-Octadecenoic acid, (E)- 111528 000112-79-8 18

59 54.842 0.29 C:\Database\NIST98.L
9-Octadecenoic acid, (E)- 111528 000112-79-8 41
9-Octadecenoic acid, (E)- 111554 000112-79-8 40
1-Benzopyrylium, 2-phenyl- 84871 014051-53-7 15

60 55.220 0.28 C:\Database\NIST98.L
Octadec-9-enoic acid 13821 1000190-13-7 48
9-Octadecenoic acid, (E)- 111528 000112-79-8 47

Pyrazolo[5,1-c][1,2,4]triazine-3-c 33379 006841-01-6 25
arboxylic acid, 4-amino-, ethyl es
ter

61 55.872 0.52 C:\Database\NIST98.L
Octasiloxane, 1,1,3,3,5,5,7,7,9,9, 27373 019095-24-0 14
11,11,13,13,15,15-hexadecamethyl-
11-Octadecenoic acid, methyl ester 13254 001937-63-9 12
, (Z)-
9-Octadecenoic acid (Z)-, 2-hydrox 13017 004500-01-0 11
yethyl ester

170223PL.M Sat Mar 11 11:52:50 2023

Polvo 1

toxi Library Search Report

Data Path : D:\vsk\MARZO\

Data File : 230314-06.D

Acq On : 10 Mar 2023 13:58 (#1); 10 Mar 2023 13:51 (#2)

Operator : KELVIN/KAREN/ALE

Sample : POLVO 1

Misc : POLVO 1 (Sig #1); (Sig #2)

ALS Vial : 4 Sample Multiplier: 1

Search Libraries: C:\Database\NIST98.L Minimum Quality: 0

C:\Database\WILEY275.L Minimum Quality: 0

Unknown Spectrum: Apex

Integration Events: ChemStation Integrator - autoint1.e

Pk# RT Area% Library/ID Ref# CAS# Qual

---

1 7.745 0.03 C:\Database\NIST98.L

Toluene 117282 000108-88-3 64

Toluene 117292 000108-88-3 50

1,3,5-Cycloheptatriene 117284 000544-25-2 45

2 8.540 0.03 C:\Database\NIST98.L

Cyclotrisiloxane, hexamethyl- 84874 000541-05-9 83

Cyclotrisiloxane, hexamethyl- 126480 000541-05-9 83

Cyclotrisiloxane, hexamethyl- 126481 000541-05-9 74

3 12.374 0.14 C:\Database\NIST98.L

Tricyclo[2.2.1.02,6]heptane, 1,3,3 117890 000488-97-1 91

-trimethyl-

4-Carene 39884 1000150-36-1 87

1S-.alpha.-Pinene 40167 007785-26-4 86

4 13.364 0.24 C:\Database\NIST98.L

Cyclotetrasiloxane, octamethyl- 97367 000556-67-2 86

Cyclotetrasiloxane, octamethyl- 128141 000556-67-2 72

3,6-Bis(N-dimethylamino)-9-ethylca 97346 057103-04-5 64

rbazole

5 13.845 0.05 C:\Database\NIST98.L

Bicyclo[3.1.0]hex-2-ene, 4-methyl- 40029 028634-89-1 86

1-(1-methylethyl)-

.beta.-Phellandrene 117859 000555-10-2 86

.beta.-Phellandrene 40031 000555-10-2 86

6 18.273 8.71 C:\Database\NIST98.L

Cyclopentasiloxane, decamethyl- 103462 000541-02-6 91

Cyclopentasiloxane, decamethyl- 128838 000541-02-6 90

Silane, [[4-[1,2-bis[(trimethylsil 103450 056114-62-6 32

yl)oxy]ethyl]-1,2-phenylene]bis(ox

y)]bis[trimethyl-

7 20.923 0.04 C:\Database\NIST98.L

Pentasiloxane, dodecamethyl- 97318 000141-63-9 86

3-Ethoxy-1,1,1,5,5,5-hexamethyl-3- 97306 018030-67-6 38

(trimethylsiloxy)trisiloxane

Cyclotetrasiloxane, octamethyl- 128141 000556-67-2 25

8 23.257 14.07 C:\Database\NIST98.L

1,3,5,7,9,11-Hexaethylbicyclo[5.5. 106141 073113-17-4 16

1]hexasiloxane

Estra-1,3,5(10)-trien-17-one, 2-[(106140 077883-26-2 14

trimethylsilyl)amino]-3-[(trimethy

lsilyl)oxy]-

Morphinan, 7,8-didehydro-4,5-epoxy 129063 055449-66-6 10

-17-methyl-3,6-bis[(trimethylsilyl

)oxy]-, (5.alpha.,6.alpha.)-

9 24.556 0.03 C:\Database\NIST98.L

Cyclopentasiloxane, decamethyl- 115315 000541-02-6 35

Cyclopentasiloxane, decamethyl- 128838 000541-02-6 25

Hexasiloxane, 1,1,3,3,5,5,7,7,9,9, 27715 000995-82-4 25

11,11-dodecamethyl-

10 25.025 0.03 C:\Database\NIST98.L

Boron, bis[.mu.-(4-bromo-1H-pyrazo 105432 014695-71-7 17

lato-N1:N2)]tetraethyldi-

Diacetylhaemanthadine 9985 1000212-96-8 9

2,4a-Oxymethano-1,2,3,4,4a,5,6,7,8 105451 1000194-82-6 9
,8a,9-dodecahydrophenanthren-9-one
, 8-cyanomethyl-2-methoxy-7-methox
ycarbonyl-1,1,7-trimethyl-

11 25.494 0.04 C:\Database\NIST98.L

2,6-Dimethyl-3,4-bis(trimethylsily 87845 1000079-52-1 32
loxymethyl)pyridine
Gibb-3-ene-1,10-dicarboxylic acid, 97331 005508-47-4 14
2,4a-dihydroxy-1-methyl-8-methyle
ne-, 1,4a-lactone, 10-methyl ester
, (1.alpha.,2.beta.,4a.alpha.,4b.b
eta.,10.beta.)-
Pentasiloxane, dodecamethyl- 97318 000141-63-9 14

12 27.308 0.43 C:\Database\NIST98.L

Comentado [MH1]: 8

Bicyclo[7.2.0]undec-4-ene, 4,11,11 108830 000118-65-0 90
-trimethyl-8-methylene-,[1R-(1R*,4
Z,9S*)]-
Caryophyllene 117850 000087-44-5 74
Bicyclo[7.2.0]undec-4-ene, 4,11,11 117804 013877-93-5 62
-trimethyl-8-methylene-

13 27.732 9.32 C:\Database\NIST98.L

Cycloheptasiloxane, tetradecamethy 27714 000107-50-6 93
l-
3,5-Dibutoxy-1,1,1,7,7,7-hexamethy 25310 072439-85-1 14

l-3,5-bis(trimethylsiloxy)tetrasil
oxane
3-Isopropoxy-1,1,1,7,7,7-hexamethy 26784 071579-69-6 12
l-3,5,5-tris(trimethylsiloxy)tetra
siloxane

14 28.870 0.07 C:\Database\NIST98.L
Germacrene D 71000 023986-74-5 91
.alpha.-Cubebene 119368 017699-14-8 59
Tetracyclo[6.1.0.0(2,4).0(5,7)]non 70883 051898-92-1 59
ane,3,3,6,6,9,9-hexamethyl-(1.alph
a.,2.alpha.,4.alpha.,5.beta.,7.bet
a.,8.alpha.)-

15 29.202 0.07 C:\Database\NIST98.L
Phenol, 3,5-bis(1,1-dimethylethyl) 125804 001138-52-9 27
2,5,5,8a-Tetramethyl-4-methylene-4 80580 1000194-97-6 22
a,5,6,7,8,8a-hexahydro-4H-chromene
1-(3,6,6-Trimethyl-1,6,7,7a-tetrah 80610 054686-01-0 22
ydrocyclopenta[c]pyran-1-yl)ethano
ne

16 30.164 0.07 C:\Database\NIST98.L
2(4H)-Benzofuranone, 5,6,7,7a-tetr 120085 017092-92-1 47
ahydro-4,4,7a-trimethyl-, (R)-
2(4H)-Benzofuranone, 5,6,7,7a-tetr 50181 017092-92-1 46
ahydro-4,4,7a-trimethyl-, (R)-

2(4H)-Benzofuranone, 5,6,7,7a-tetr 120127 015356-74-8 46

ahydro-4,4,7a-trimethyl-

17 31.314 0.11 C:\Database\NIST98.L

1H-Cycloprop[e]azulen-7-ol, decahy 4517 006750-60-3 40

dro-1,1,7-trimethyl-4-methylene-,

[1ar-(1a.alpha.,4a.alpha.,7.beta.,

7a.beta.,7b.alpha.)]-

(-)-Spathulenol 4516 077171-55-2 38

Spathulenol 4610 1000153-10-5 32

18 31.485 0.29 C:\Database\NIST98.L

Caryophyllene oxide 109343 001139-30-6 90

Caryophyllene oxide 4472 1000156-32-9 86

Bicyclo[6.1.0]nonane, 9-(1-methyle 2380 056666-90-1 53

thylidene)-

19 31.697 6.32 C:\Database\NIST98.L

Silane, [[4-[1,2-bis[(trimethylsil 128841 056114-62-6 50

yl)oxy]ethyl]-1,2-phenylene]bis(ox

y)]bis[trimethyl-

1,3,5,7-Tetraethyl-1-ethylbutoxysi 103472 073420-30-1 46

loxycyclotetrasiloxane

Silane, [[4-[1,2-bis[(trimethylsil 103450 056114-62-6 43

yl)oxy]ethyl]-1,2-phenylene]bis(ox

y)]bis[trimethyl-

20 32.773 0.15 C:\Database\NIST98.L

3-Methylene-bicyclo[3.2.1]oct-6-en 61786 1000193-00-0 30

-8-ol

10,10-Dimethyl-2,6-dimethylenebicy 61706 019431-80-2 27

clo[7.2.0]undecan-5.beta.-ol

2H-Inden-2-one, 1,4,5,6,7,7a-hexah 61794 039163-29-6 27

ydro-

21 33.482 0.09 C:\Database\NIST98.L

1H-Indene, 1-ethylideneoctahydro-7 66731 056324-69-7 27

a-methyl-, (1Z,3a.alpha.,7a.beta.)

Propane, 1-(2,2-dichloro-1,3,3-tri 1257 024551-93-7 22

methylcyclopropyl)-

1H-3a,7-Methanoazulene, octahydro- 14079 025491-20-7 14

1,4,9,9-tetramethyl-

22 33.826 0.08 C:\Database\NIST98.L

1H-Cycloprop[e]azulen-7-ol, decahy 4517 006750-60-3 43

dro-1,1,7-trimethyl-4-methylene-,

[1ar-(1a.alpha.,4a.alpha.,7.beta.,

7a.beta.,7b.alpha.)]-

Tricyclo[4.4.0.02,7]dec-3-ene-3-me 38321 115728-41-1 38

thanol, 1-methyl-8-(1-methylethyl)

7-Tetracyclo[6.2.1.0(3.8)0(3.9)]un 70310 1000188-90-7 35

decanol, 4,4,11,11-tetramethyl-

23 35.113 5.27 C:\Database\NIST98.L

4-(3,4-Dimethoxybenzylidene)-1-(4- 106126 1000224-94-9 86

nitrophenyl)-3-phenyl-2-pyrazolin-

5-one

Cyclononasiloxane, octadecamethyl- 115158 000556-71-8 74

2-(5-Bromo-3-pyridyl)-5-[(5-chloro 106137 1000225-99-3 35

salicylidene)amino]benzoxazole

24 36.635 0.16 C:\Database\NIST98.L

1-Methoxy-3-(2-hydroxyethyl)nonane 11495 1000216-82-6 50

11-Hexadecen-1-ol, acetate, (Z)- 13558 034010-21-4 47

1,4-Eicosadiene 21951 1000131-16-3 45

25 36.767 0.06 C:\Database\NIST98.L

Tricyclo[7.3.0.0(3,8)]dodecan-12-o 56631 1000161-32-5 38

ne, (Z)-1,9-cisoid-8,9-(Z)-3,8-11-

(but-3-en-1-yl)-2.alpha.-cyano-2.b

eta.-methyl-9.beta.-methyl-

2-(4-Methoxyphenoxy)-N-(2,4-xylyl) 97849 1000224-62-9 30

acetamide

Silane, (bromomethyl)- 4739 007570-21-0 27

26 37.144 0.05 C:\Database\NIST98.L

Cyclohexanol, 5-methyl-2-(1-methyl 24620 015356-70-4 38

ethyl)-, (1.alpha.,2.beta.,5.alpha

.)-(.+/-.)-

Z-11(13,14-Epoxy)tetradecen-1-ol a 7514 1000131-33-2 30

cetate

2,2-Dimethylocta-3,4-dienal 55880 000590-71-6 27

27 37.539 0.06 C:\Database\NIST98.L

1,19-Eicosadiene 13201 014811-95-1 38

18-Nonadecen-1-ol 13304 1000142-89-2 35

Cyclopentaneethanol, 2-(hydroxymet 31367 000485-42-7 27

hyl)-.beta.,3-dimethyl-

28 38.140 4.73 C:\Database\NIST98.L

Cyclodecasiloxane, eicosamethyl- 27712 018772-36-6 90

1,1,1,5,7,7,7-Heptamethyl-3,3-bis(26950 038147-00-1 35

trimethylsiloxy)tetrasiloxane

Octasiloxane, 1,1,3,3,5,5,7,7,9,9, 27373 019095-24-0 27

11,11,13,13,15,15-hexadecamethyl-

29 38.277 0.04 C:\Database\NIST98.L

7,9-Di-tert-butyl-1-oxaspiro(4,5)d 17969 1000143-92-4 58

eca-6,9-diene-2,8-dione

Arteannuin b 4211 1000212-12-6 30

Pyrrolidin-5-one, 2,3-dedihydro-3- 14732 1000124-44-1 18

nitro-

30 38.832 0.06 C:\Database\NIST98.L

Thianthrene 126750 000092-85-3 72

Thianthrene 126748 000092-85-3 64

Thianthrene 86759 000092-85-3 59

31 40.726 0.24 C:\Database\NIST98.L

Hexadecanoic acid, trimethylsilyl 128570 055520-89-3 38

ester

Benzene, (2-methyl-2-propenyl)- 52561 003290-53-7 11

Benzene, 2-butenyl- 120542 001560-06-1 11

32 40.898 4.47 C:\Database\NIST98.L

Cyclodecasiloxane, eicosamethyl- 27712 018772-36-6 60

Silane, [[4-[1,2-bis[(trimethylsil 128842 056114-62-6 35

yl)oxy]ethyl]-1,2-phenylene]bis(ox

y)]bis[trimethyl-

Benzeneacetic acid, .alpha.,3,4-tr 103445 055268-65-0 30

is[(trimethylsilyl)oxy]-, methyl e

ster

33 42.105 0.13 C:\Database\NIST98.L

Phytol 114546 000150-86-7 58

Phytol 114589 000150-86-7 53

Phytol 24413 000150-86-7 53

34 42.660 0.08 C:\Database\NIST98.L

Heptasiloxane, 1,1,3,3,5,5,7,7,9,9 27374 019095-23-9 40

,11,11,13,13-tetradecamethyl-

Perhydro-htx-2-one, 2-depentyl-, a 9964 1000126-27-9 11

cetate ester

n-Nonadecanoic acid, pentamethyldi 10264 1000217-02-3 10

silyl ester

35 43.387 4.80 C:\Database\NIST98.L

2-(5-Bromo-3-pyridyl)-5-[(5-chloro 106137 1000225-99-3 38

salicylidene)amino]benzoxazole

4-(3,4-Dimethoxybenzylidene)-1-(4- 106126 1000224-94-9 38

nitrophenyl)-3-phenyl-2-pyrazolin-

5-one

Estra-1,3,5(10)-trien-17-one, 2-[(106140 077883-26-2 25

trimethylsilyl)amino]-3-[(trimethy

lsilyl)oxy]-

36 43.902 0.26 C:\Database\NIST98.L

trans-9-Octadecenoic acid, trimeth 25978 096851-47-7 96

ylsilyl ester

Oleic acid, trimethylsilyl ester 120456 021556-26-3 95

11-cis-Octadecenoic acid, trimethy 25916 1000079-02-6 93

lsilyl ester

37 44.022 0.02 C:\Database\NIST98.L

Oleic acid TMS 25974 1000141-48-4 25

Cyclopentanemethanol, .alpha.-cycl 41416 103077-58-3 14

ohexyl-2-nitro-

Docosanoic acid 109677 000112-85-6 14

38 44.657 1.59 C:\Database\NIST98.L

2,2':5',2''-Terthiophene 92802 001081-34-1 98

2,2':4',2''-Terthiophene 92714 021732-47-8 95

2,2':5',2''-Terthiophene 127600 001081-34-1 94

39 45.012 0.05 C:\Database\NIST98.L

2-(3-Acetoxy-4,4,10,13,14-pentamet 103434 1000194-01-2 41
hyl-2,3,4,5,6,7,10,11,12,13,14,15,
16,17-tetradecahydro-1H-cyclopenta
[a]phenanthren-17-yl)-propioni
1,3,5,7,9,11-Hexaethylcyclohexasil 103486 017002-87-8 38
oxane
Octasiloxane, 1,1,3,3,5,5,7,7,9,9, 27373 019095-24-0 32
11,11,13,13,15,15-hexadecamethyl-

40 45.687 5.60 C:\Database\NIST98.L

Cyclononasiloxane, octadecamethyl- 115158 000556-71-8 38
4-(3,4-Dimethoxybenzylidene)-1-(4- 106126 1000224-94-9 25
nitrophenyl)-3-phenyl-2-pyrazolin-
5-one
Pentasiloxane, dodecamethyl- 115143 000141-63-9 18

41 46.408 0.07 C:\Database\NIST98.L

Furan, 2-ethoxy-5-ethyl-4-phenyl- 86762 1000137-69-6 47
Cyclobutane, tetrakis(1-methylethy 86729 088919-66-8 47
lidene)-
Pyrene, 1,2,3,3A,4,5,5A,6,7,8,8A,9 86763 126188-35-0 43
,10,10A-tetradecahydro-

42 47.209 0.04 C:\Database\NIST98.L

Hexasiloxane, 1,1,3,3,5,5,7,7,9,9, 27715 000995-82-4 25

11,11-dodecamethyl-

Tricyclo[10.2.2.25,8]octadeca-5,7, 52694 024777-32-0 12

12,14,15,17-hexaene, 6-nitro-

Octasiloxane, 1,1,3,3,5,5,7,7,9,9, 27373 019095-24-0 10

11,11,13,13,15,15-hexadecamethyl-

43 47.850 6.48 C:\Database\NIST98.L

4-(3,4-Dimethoxybenzylidene)-1-(4- 106126 1000224-94-9 53

nitrophenyl)-3-phenyl-2-pyrazolin-

5-one

Cyclononasiloxane, octadecamethyl- 115158 000556-71-8 41

Benzoic acid, 2,5-bis(trimethylsil 103449 003618-20-0 38

oxy)-, trimethylsilyl ester

44 48.371 0.08 C:\Database\NIST98.L

Butyl oleate 2000 1000131-35-1 55

E,Z-1,3,12-Nonadecatriene 21430 1000131-11-3 45

1,3,12-Nonadecatriene 21143 1000131-11-1 41

45 48.697 0.08 C:\Database\NIST98.L

2(3H)-Furanone, dihydro-5-tetradec 116682 000502-26-1 90

yl-

Tricyclo[10.2.2.25,8]octadeca-5,7, 52694 024777-32-0 38

12,14,15,17-hexaene, 6-nitro-

Eicosane 112565 000112-95-8 25

46 49.281 0.09 C:\Database\NIST98.L

Benzoic acid, 5-methyl-2-trimethyl 97301 1000153-59-4 25

silyloxy-, trimethylsilyl ester

Benzoic acid, 4-methyl-2-trimethyl 97300 1000153-59-3 25

silyloxy-, trimethylsilyl ester

6H-Dibenzo[a,g]quinolizine, 5,8,13 67163 002934-97-6 25

,13a-tetrahydro-2,3,9,10-tetrameth

oxy-, (.+/-.)-

47 49.887 7.27 C:\Database\NIST98.L

4-(3,4-Dimethoxybenzylidene)-1-(4- 106126 1000224-94-9 53

nitrophenyl)-3-phenyl-2-pyrazolin-

5-one

Cyclononasiloxane, octadecamethyl- 115158 000556-71-8 50

Benzoic acid, 2,5-bis(trimethylsil 103449 003618-20-0 44

oxy)-, trimethylsilyl ester

48 49.996 0.26 C:\Database\NIST98.L

4-O-Methoxyphenylhydrazono-3-methy 89950 1000148-00-4 70

l-2-pyrazolin-5-one

Phenoxathiin, 10,10-dioxide 89951 000950-47-0 43

Acetic acid, 3-methoxy-7-methyl-6, 90006 1000189-21-8 40

7,8,9-tetrahydro-dibenzofuran-2-yl

ester

49 51.060 0.07 C:\Database\NIST98.L

Elaidic acid, isopropyl ester 13539 022147-34-8 91

Dodecahydropyrido[1,2-b]isoquinoli 84738 1000195-30-7 42

n-6-one

Hexahydropyridine, 1-methyl-4-[4,5 84723 094427-47-1 27

-dihydroxyphenyl]-

50 51.215 0.09 C:\Database\NIST98.L

Octasiloxane, 1,1,3,3,5,5,7,7,9,9, 27373 019095-24-0 64

11,11,13,13,15,15-hexadecamethyl-

Heptasiloxane, 1,1,3,3,5,5,7,7,9,9 27374 019095-23-9 59

,11,11,13,13-tetradecamethyl-

Cyclononasiloxane, octadecamethyl- 115158 000556-71-8 22

51 51.306 0.07 C:\Database\NIST98.L

Butane, 2-phenyl-3-(trimethylsilyl 52301 1000163-21-4 38

oxy)-

Benzene, 1-[(4-ethoxyphenyl)ethyny 84924 039969-29-4 22

l]-4-propyl-

cis, 6-Octadecenoic acid, trimethy 26547 096851-53-5 14

lsilyl ester

52 51.352 0.06 C:\Database\NIST98.L

2,6-Dimethyl-5-methylphenylaminopy 97353 1000222-06-1 18

ridin-3,4-dicarboxyimide

Cyclopentanecarboxamide, 3-ethenyl 97312 136091-23-1 15

-2-(3-pentenylidene)-N-phenyl-, [1

.alpha.,2Z(E),3.alpha.]-

3-Isopropoxy-1,1,1,5,5,5-hexamethy 27710 072182-11-7 14

l-3-(trimethylsiloxy)trisiloxane

53 51.587 0.06 C:\Database\NIST98.L

Propyleneglycol monoleate 21252 1000132-46-8 42

Benzene, 2-[(tert-butyldimethylsil 84728 1000221-75-3 22

yl)oxy]-1-isopropyl-4-methyl-

Dodecahydropyrido[1,2-b]isoquinoli 84738 1000195-30-7 18

n-6-one

54 51.638 0.06 C:\Database\NIST98.L

Hexahydropyridine, 1-methyl-4-[4,5 84723 094427-47-1 45

-dihydroxyphenyl]-

Dodecahydropyrido[1,2-b]isoquinoli 84738 1000195-30-7 22

n-6-one

1H-Indole, 2-methyl-3-phenyl- 126477 004757-69-1 22

55 51.684 0.06 C:\Database\NIST98.L

Benzene, 1-[(4-ethoxyphenyl)ethyny 84924 039969-29-4 25

l]-4-propyl-

Pyridine, 1,2,3,6-tetrahydro-1-met 84703 005048-08-8 18

hyl-4-[4-chlorophenyl]-

9-Octadecenoic acid (Z)-, 2-hydrox 13221 003443-84-3 15

y-1-(hydroxymethyl)ethyl ester

56 51.821 8.28 C:\Database\NIST98.L

Cyclononasiloxane, octadecamethyl- 115158 000556-71-8 64

Silane, [[4-[1,2-bis[(trimethylsil 128842 056114-62-6 27

yl)oxy]ethyl]-1,2-phenylene]bis(ox
y)]bis[trimethyl-
4-(3,4-Dimethoxybenzylidene)-1-(4- 106126 1000224-94-9 25
nitrophenyl)-3-phenyl-2-pyrazolin-
5-one

57 51.913 0.47 C:\Database\NIST98.L
Octasiloxane, 1,1,3,3,5,5,7,7,9,9, 27373 019095-24-0 30
11,11,13,13,15,15-hexadecamethyl-
Tricyclo[10.2.2.25,8]octadeca-5,7, 52694 024777-32-0 22
12,14,15,17-hexaene, 6-nitro-
Benzene, 2-[(tert-butyldimethylsil 84728 1000221-75-3 14
yl)oxy]-1-isopropyl-4-methyl-

58 52.588 0.16 C:\Database\NIST98.L
Cyclopentene-1-carboxylic acid, 3- 84769 1000159-40-6 32
[2-(diphenylmethyl)-2-propen-1-yl]
-, methyl ester
Acetic acid, [4-(1,1-dimethylethyl 84742 088530-52-3 11
)phenoxy]-, methyl ester
1H-Indole, 2-methyl-3-phenyl- 126477 004757-69-1 11

59 52.657 0.08 C:\Database\NIST98.L
cis-4-Ethoxy-b-methyl-b-nitrostyre 84793 1000120-36-6 38
ne
trans-3-Ethoxy-b-methyl-b-nitrosty 84753 023037-46-9 38
rene

Heptasiloxane, 1,1,3,3,5,5,7,7,9,9 27374 019095-23-9 25

,11,11,13,13-tetradecamethyl-

60 52.817 0.05 C:\Database\NIST98.L

Hexahydropyridine, 1-methyl-4-[4,5 84723 094427-47-1 22

-dihydroxyphenyl]-

1H-Indole, 2-methyl-3-phenyl- 126477 004757-69-1 18

3,3,7,11-Tetramethyltricyclo[5.4.0 84762 1000140-22-7 15

.0(4,11)]undecan-1-ol

61 53.189 0.11 C:\Database\NIST98.L

Octasiloxane, 1,1,3,3,5,5,7,7,9,9, 27373 019095-24-0 50

11,11,13,13,15,15-hexadecamethyl-

2,3,5,5,8a-Pentamethyl-6,7,8,8a-te 9562 1000190-30-5 43

trahydro-5H-chromen-8-ol

1,4-Benzenediol, 2,5-bis(1,1-dimet 84896 000088-58-4 43

hylethyl)-

62 53.343 0.02 C:\Database\NIST98.L

Benzene, 2-[(tert-butyldimethylsil 84728 1000221-75-3 38

yl)oxy]-1-isopropyl-4-methyl-

Hexahydropyridine, 1-methyl-4-[4,5 84723 094427-47-1 35

-dihydroxyphenyl]-

Dodecahydropyrido[1,2-b]isoquinoli 84738 1000195-30-7 20

n-6-one

63 53.366 0.04 C:\Database\NIST98.L

Silane, trimethyl[5-methyl-2-(1-me 126457 055012-80-1 15

thylethyl)phenoxy]-

Silanamine, N-[2,6-dimethyl-4-[(tr 27716 072088-09-6 11

imethylsilyl)oxy]phenyl]-1,1,1-tri

methyl-

Propanamide, N-(3-methoxyphenyl)-2 17665 056619-93-3 11

,2-dimethyl-

64 53.698 0.26 C:\Database\NIST98.L

2,6,10-Dodecatrienoic acid, 3,7,11 23047 020085-73-8 47

-trimethyl-, ethyl ester, (Z,Z)-

Cyclopentane, 1,1'-[3-(2-cyclopent   2576 055255-85-1 40

ylethyl)-1,5-pentanediyl]bis-

2,4,4-Trimethyl-3b-hydroxymethyl-5 23272 1000144-10-5 38

a-(3-methyl-but-2-enyl)-cyclohexen

65 53.893 7.45 C:\Database\NIST98.L

Cyclononasiloxane, octadecamethyl- 115158 000556-71-8 62

Cyclodecasiloxane, eicosamethyl-   27712 018772-36-6 27

Trisiloxane, 1,1,1,5,5,5-hexamethy 26961 003555-47-3 25

l-3,3-bis[(trimethylsilyl)oxy]-

66 55.232 0.05 C:\Database\NIST98.L

1,2-Dihydro(4H)anthra(1,2-d)(1,3)o 95151 1000098-77-3 25

xazine-7,12-dione

Benzene, 1-[(4-ethoxyphenyl)ethyny 84924 039969-29-4 25

l]-4-propyl-

Methadone N-oxide 84766 1000120-80-7 22

67 55.449 0.09 C:\Database\NIST98.L

Octasiloxane, 1,1,3,3,5,5,7,7,9,9, 27373 019095-24-0 58

11,11,13,13,15,15-hexadecamethyl-

Heptasiloxane, 1,1,3,3,5,5,7,7,9,9 27374 019095-23-9 49

,11,11,13,13-tetradecamethyl-

3,3,7,11-Tetramethyltricyclo[5.4.0 84762 1000140-22-7 38

.0(4,11)]undecan-1-ol

68 55.718 0.05 C:\Database\NIST98.L

1H-Indole, 2-methyl-3-phenyl- 126477 004757-69-1 30

Ethyl 2-(2-chloroacetamido)-3,3,3- 84746 1000224-17-1 27

trifluoro-2-(3-fluoroanilino)propi

onate

Isolongifolan-8-ol 3146 001139-08-8 22
