# Supplementary figures and images for: From Gas Chromatography–Mass Spectrometry (GC–MS) to Network Pharmacology: System-Level Insights into the Multi-Target Biological Potential of Flaveria trinervia (Spreng.) C. Mohr
Source: Curr Issues Mol Biol. 2026 Feb 1;48(2):160. doi: 10.3390/cimb48020160 (PMC12939618; doi:10.3390/cimb48020160)

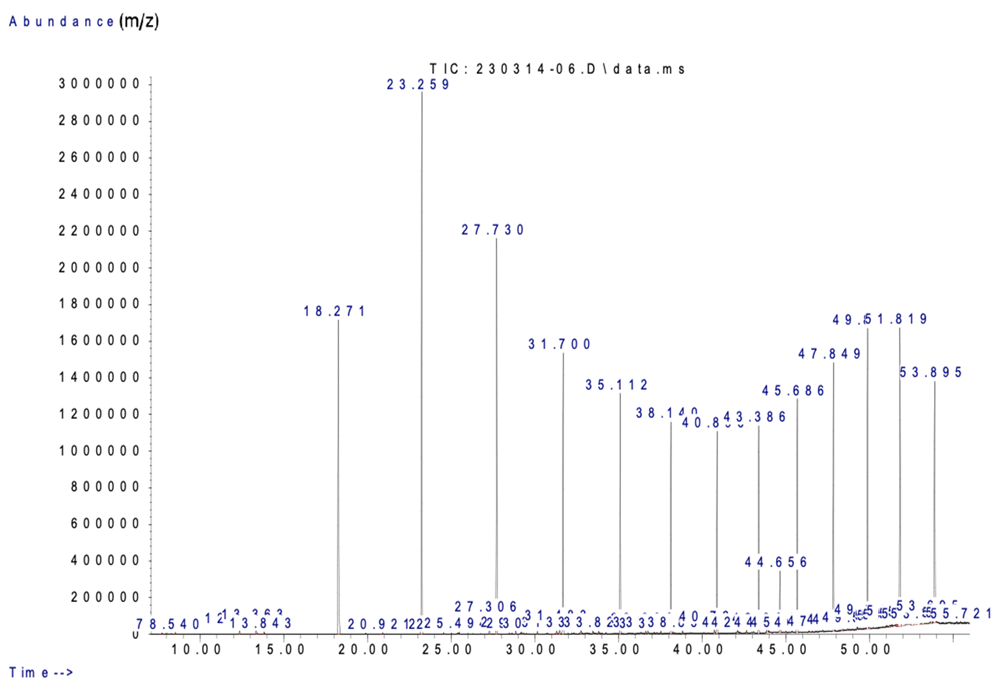

Supplement: Supplementary file 1 [file cimb-48-00160-s001.zip › Material suplementario/Figure 1A.png]

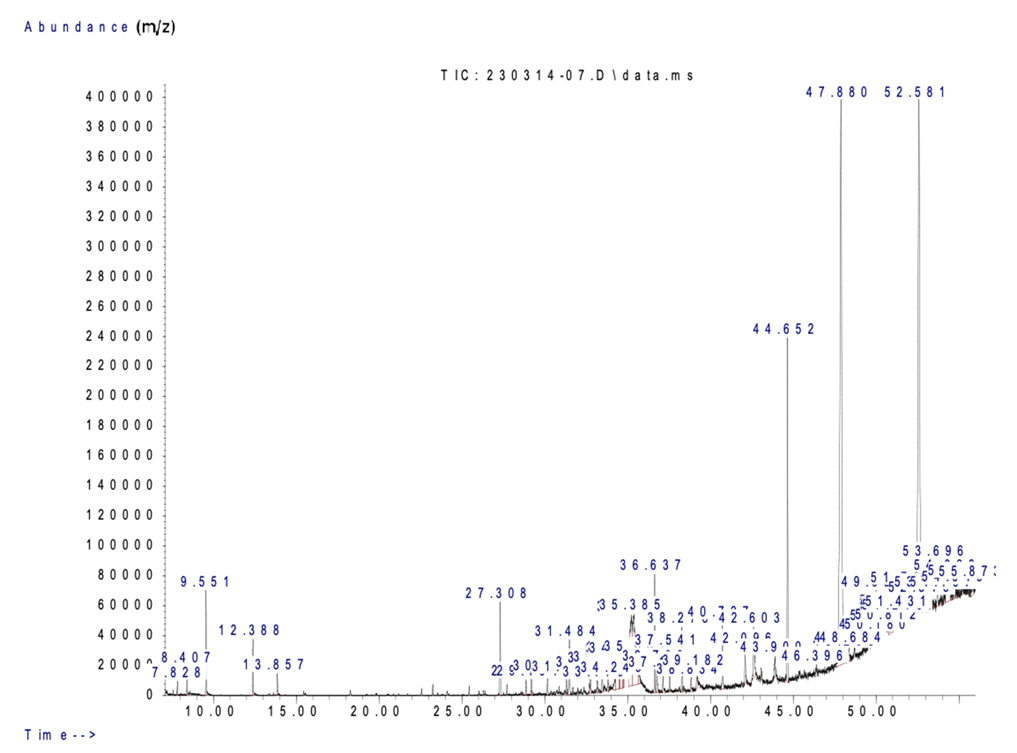

Supplement: Supplementary file 1 [file cimb-48-00160-s001.zip › Material suplementario/Figure 1B.png]

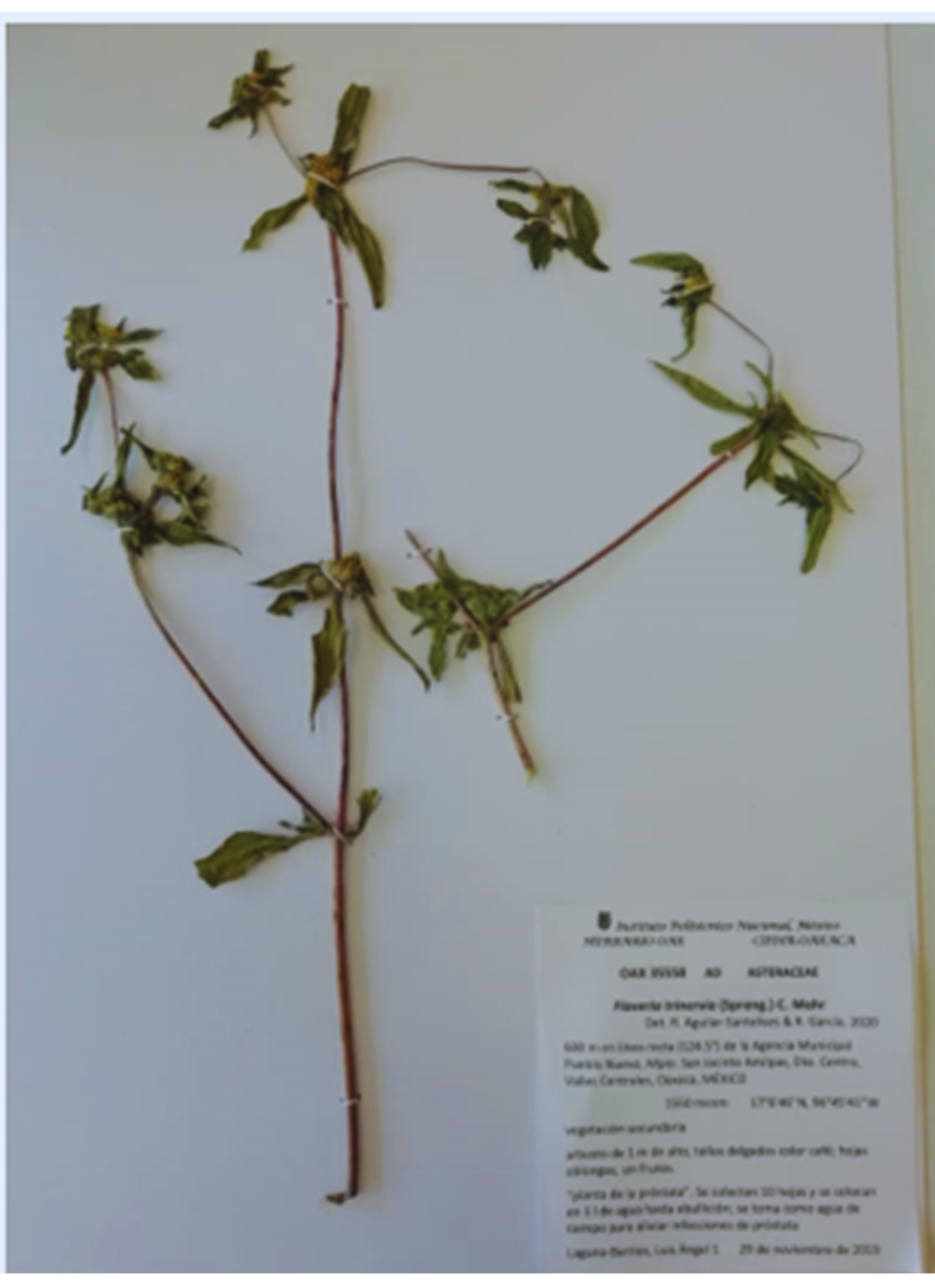

Supplement: Supplementary file 1 [file cimb-48-00160-s001.zip › Material suplementario/Phenological stage F. t.png]
